# Environmentally driven transcriptomic and metabolic changes leading to color differences in “Golden Reinders” apples

**DOI:** 10.3389/fpls.2022.913433

**Published:** 2022-08-01

**Authors:** Pablo Fernández-Cancelo, Ariadna Iglesias-Sanchez, Salvador Torres-Montilla, Albert Ribas-Agustí, Neus Teixidó, Manuel Rodriguez-Concepcion, Jordi Giné-Bordonaba

**Affiliations:** ^1^Postharvest Programme, Institute of Agrifood Research and Technology (IRTA), Lleida, Spain; ^2^Centre for Research in Agricultural Genomics (CRAG) CSIC-IRTA-UAB-UB, Barcelona, Spain; ^3^Institute for Plant Molecular and Cell Biology (IBMCP), CSIC-Universitat Politècnica de València, Valencia, Spain; ^4^Food Safety and Functionality Programme, IRTA, Monells, Spain

**Keywords:** antioxidant metabolism, color, environment, isoprenoids, oxidative stress, phenylpropanoids, *Malus domestica* (Bork.)

## Abstract

Apple is characterized by its high adaptation to diverse growing environments. However, little is still known about how different environments can regulate at the metabolic or molecular level specific apple quality traits such as the yellow fruit peel color. In this study, changes in carotenoids and chlorophylls, antioxidants as well as differences in the transcriptome were investigated by comparing the peel of “Golden Reinders” apples grown at different valley and mountain orchards. Mountain environment favored the development of yellow color, which was not caused by an enhanced accumulation of carotenoids but rather by a decrease in the chlorophyll content. The yellow phenotype was also associated to higher expression of genes related to chloroplast functions and oxidative stress. Time-course analysis over the last stages of apple development and ripening, in fruit from both locations, further revealed that the environment differentially modulated isoprenoids and phenylpropanoid metabolism and pointed out a key role for H_2_O_2_ in triggering apple peel degreening. Overall, the results presented herein provide new insights into how different environmental conditions regulate pigment and antioxidant metabolism in apple leading to noticeable differences in the apple peel color.

## Introduction

Fruit peel color is an attribute highly appreciated by consumers (Iglesias et al., 2008) which is controlled by the relative concentrations of pigments, including isoprenoids (chlorophylls and carotenoids) and phenylpropanoids (anthocyanins; Merzlyak et al., 2003). Chlorophylls and carotenoids are both photosynthetic pigments associated with light-harvesting proteins which are involved in light absorption processes within the thylakoids (Polívka and Frank, 2010). While chlorophylls are responsible for the green color in unripe apples (Reay et al., 1998), carotenoids are the major pigments in yellow peel apples such as “Golden Delicious” (Delgado-Pelayo et al., 2014). During ripening, but also as a response to stress, chlorophyll levels decrease accompanying the perceived fruit color changes (Yin et al., 2016). Carotenoids are synthetized and stored in plastids, including chloroplasts, where they are required for the assembly of the photosynthetic apparatus and are essential for photoprotection by scavenging reactive oxygen species (ROS) and by dissipating excess light energy as heat (Rodriguez-Concepcion et al., 2018). Carotenoids also act together with tocopherols (another group of plastidial isoprenoids) as thylakoid antioxidants (Ramel et al., 2012). In addition, carotenoids are the precursors of abscisic acid (ABA), a hormone involved in the regulation of fruit ripening (Leng et al., 2013) and in the fruit or plant adaptation to environmental conditions (Wang et al., 2020). As an example, ABA is able to orchestrate the fruit tolerance to abiotic stresses by regulating the accumulation of ROS (Zhang et al., 2019) and the expression of antioxidant enzymes (Mou et al., 2015), such as superoxide dismutase (SOD), catalase (CAT), and peroxidases (APX and POX; Meitha et al., 2020). Indeed, antioxidant enzymes in combination with non-enzymatic antioxidants, such as phenolic compounds, tocopherols, carotenoids, ascorbic acid, and glutathione (Shao et al., 2008), are able to limit the accumulation of damaging ROS produced either during aerobic metabolism (Venditti et al., 2013) or under stress conditions (Choudhury et al., 2017). ROS (i.e., H_2_O_2_) not only act as damaging but also as signaling molecules participating in transduction cascades (Saxena et al., 2016), interacting with plant hormones (Chen et al., 2016), and regulating physiological processes such as fruit development (Khandaker et al., 2012). Some studies suggest that H_2_O_2_ may regulate, for instance, the chloroplast-to-chromoplast transition in carotenoid-rich fruit during ripening (Bouvier et al., 1998), favoring carotenoids accumulation and color changes (Sadali et al., 2019). However, in “Golden” apples, carotenoid levels do not appear to increase but to decrease during fruit growth (Gross et al., 1978), suggesting that other mechanisms are likely involved in color development within this particular apple cultivar.

The fruit pigment content is strongly influenced by the environment, mainly temperature (Lin-Wang et al., 2011) and light (Llorente et al., 2016). For example, it has been observed that growing “Fuji” apples at high altitudes, where temperatures are low and sunlight incidence is high, promotes the accumulation of anthocyanins (Karagiannis et al., 2020). Anthocyanins own different roles within the fruit, including the protection against photo-oxidative stress induced by high light incidence (Zheng et al., 2020) and contributing, as non-photosynthetic pigments, to the red fruit color. Nowadays, and as a result of climate change, the environmental conditions in certain production areas are no longer suitable for developing fruit with optimal peel color (Lin-Wang et al., 2011), or other optimum quality traits (Sugiura et al., 2013). For instance, a recent study demonstrated that “Golden Reinders” apples grown in the Ebro valley never attained the yellow phenotype as observed in fruit grown in mountain regions, even if the fruit was left on the tree far beyond the commercial harvest date (Fernández-Cancelo et al., 2022). This said, no information is currently available on how the environment influences, at the metabolic and transcriptomic level, yellow peel color in “Golden” apples, despite being one of the most widely cultivated and consumed varieties worldwide (Bonany et al., 2013).

Accordingly, our study aimed to decipher the biochemical and transcriptomic shifts leading to color differences in the peel of “Golden Reinders” apples grown in different environments (mountain vs. valley) as well as to elucidate if color changes were only caused by differences in pigments content or rather to other metabolic shifts driven by environmental signals able to alter peel ripening. Special emphasis was also given to clarify how pigment content and antioxidant metabolism of the apple peel change during the last stages of fruit development and ripening in response to diverse growing environments.

## Materials and methods

### Plant material and experimental design

In 2018, four different orchards located in the province of Lleida (Catalonia, North-East Spain) were used to assess the influence of the environment on apple color at the commercial harvest date (CHD): two orchards from valley areas, Alcanó (214 meters above sea level, masl) and Vilanova (195 masl), and two from mountain areas, Llesp (989 masl) and Gotarta (1,191 masl). “Golden Reinders” apples were harvested at the CHD based on flesh firmness (71 ± 2.5 N) and starch index values (6 ± 0.8) from trees of similar age and grown under the same rootstock (M9) as well as similar agronomical practices. Further characterization of the fruit maturity at the time of harvest (focused on ethylene biosynthesis) is available from Fernández-Cancelo et al. (2021). A set of 45 fruit were harvested at the same canopy height from 10 random trees in each location at CHD. Thirty fruit were used for quality evaluations (color, firmness, and starch index), whereas the peel from 15 fruit (5 apples per replicate and 3 replicates per location) were grinded and frozen in liquid nitrogen and kept at −80°C until further biochemical determinations.

Based on the color differences found in 2018, the next year fruit development and ripening were monitored in Vilanova (valley) and Gotarta (mountain) orchards at 4 sampling points, including the CHD (165 and 167 days after full bloom [DAFB] in valley and mountain orchards, respectively), based on the percentage of final fruit size (S1: 60% final fruit size, S2: 80% final fruit size, S3: 90% final fruit size, and S4: 100% final fruit size). Samplings started at 89 DAFB (corresponding with S1) and were performed approximately every 25–30 days differing less than 5 days between locations for each sampling point. The final fruit size (equivalent to CHD) also corresponded to 1,388 and 848 growing degree days (GDD) for apples from Vilanova and Gotarta, respectively, within the studied period. At each harvest, 50 fruit per location and sampling point were harvested at the same canopy height from 10 random trees and immediately transported to the laboratory for quality evaluations (30 fruit per location or sampling point). In parallel, peel from 20 fruit (5 apples per replicate and 4 replicates per location and sampling point) were ground and frozen in liquid nitrogen and kept at −80°C until further biochemical analysis.

Meteorological data including temperature, relative humidity (HR), evapotranspiration, and solar radiation were recorded at Vilanova and Gotarta orchards by portable meteorological stations (Decagon Devices, Pullman, WA, United States) equipped with a pyranometer (Apogee Instruments, Logan, UT, United States).

### Fruit quality evaluation

On arrival to the laboratory, fruit weight, diameter, color, firmness, and starch index were measured on 30 individual fruit per orchard and sampling point. In the particular case of color and firmness determinations, measurements were made on two opposite sides of each examined fruit using a portable colorimeter (CM-2600d; Konica Minolta Sensing, Osaka, Japan) and a GÜSS FTA penetrometer (FR Turoni, Forlì, Italy), respectively. Starch index was evaluated by dipping equatorial fruit slices in an iodine solution (I_2_-KI) for 10 min. The starch index was assigned to each fruit using the starch scale from 1 to 10 developed by the Centre Technique Interprofessionnel des Fruits et Légumes (CTIFL, Paris, France). Further quality measurements were earlier reported by Fernández-Cancelo et al. (2021).

### RNA extraction and sequencing

RNA was extracted from lyophilized peel samples, using the Spectrum™ Plant Total RNA Kit (Sigma-Aldrich, St Louis, MO, United States) following the manufacturer’s recommendation. Electrophoresis on an agarose gel by staining with GelRed™ Nucleic Acid Gel Stain (Biotium, Hayward, CA, United States) was used for the evaluation of RNA integrity and the absence of contaminant DNA. RNA-seq service was performed by Sequentia Biotech SL (Barcelona, Spain). Assessment of RNA quality and indexed library preparation was carried out as described (Llorente et al., 2020). Sequencing was performed using the NovaSeq6000 System (Illumina) 2×150 bp, 30 M paired reads per sample. Data analysis was performed with the online platform AIR^1^ using the *Malus* × *domestica* “Golden Delicious” GDDH13 v1.1 reference genome (Daccord et al., 2017) deposited in the Genome Database from Rosacea (GDR^2^). Differentially expressed genes (DEGs) were identified with the DESeq2 statistical method in the AIR platform. The resulting lists were filtered using cut-offs of false discovery rate < 0.05 and log2-transformed fold change (log2FC) >0.585 for upregulated genes and < −0.599 for downregulated genes. GO enrichment analyses were performed using default parameters with AppleMDO^3^ automatically followed by AgriGO^4^. Enriched GO terms and *p*-values were then loaded into REVIGO^5^ to identify and visualize non-redundant GO terms using a cutoff value of 0.5.

### cDNA and qPCR analysis

The cDNA synthesis was performed on 1 μg of RNA using the SuperScript IV First-Strand Synthesis System (Invitrogen, Carlsbad, CA, United States). The gene expression analysis was performed by mixing KAPA SYBR® Fast qPCR Master Mix (Kapa Biosystems, Inc., Wilmington, MA, United States), 100 nM of each primer, and the corresponding diluted cDNA. The reaction was performed on a 7,500 Real-Time PCR System (Applied Biosystems, Waltham, MA, United States) under the following conditions: 10 s at 95°C followed by 40 cycles of 95°C during 15 s and 60°C for 1 min. A non-template control (NTC) was included by using DNA-free water instead of cDNA. A melt curve analysis was performed to check primer specific by including a final amplification cycle at 95°C for 15 s, 60°C for 1 min, 95°C for 30 s, and 60°C for 15 s. Primers used in this study were retrieved from the literature or designed *de novo* when indicated (Supplementary Table S1). Relative gene expression was expressed as Mean Normalized Expression (MNE) according to previous studies (Muller et al., 2002) using *Md8283* as a reference gene.

### Photosynthetic pigments and tocopherols analysis

Carotenoids, chlorophylls, and tocopherols were extracted from lyophilized apple peel tissue as described for tomato leaves (Barja et al., 2021). An Agilent 1,200 series HPLC system (Agilent Technologies, Santa Clara, CA, United States) was used for the separation and detection of individual species with a photodiode array for (chlorophylls and carotenoids) or a fluorescence detector (for tocopherols). Quantification was performed by comparison of peak areas with commercial standards.

### ABA quantification

ABA was extracted from lyophilized apple peel tissue as described elsewhere (Torres et al., 2017). The extract (5 μl) was injected on a Waters ACQUITY UPLC chromatograph (Waters Corporation, Milford, MA, United States) fitted with a Waters ACQUITY UPLC HSS T3 1.8 μm 100×2.1 mm column (Waters Corporation, Milford, MA, United States). Separation was carried out at a flow rate of 0.40 ml/min using a mobile phase consisting in a mixture of 0.1% formic acid and 2% of acetonitrile in water (A) and 0.1% formic acid in acetonitrile (B). The gradient program was held for the first 1 min at 5% B, increased linearly to 100% B in 1.5 min, and then maintained at 100% B for 1 min. The temperature of the column was kept at 35°C and the sample compartment was refrigerated at 10°C. Quantification was performed by comparison of peak areas with the commercial standard.

### Quantification of hydrogen peroxide and malondialdehyde

Hydrogen peroxide concentration expressed in mmol kg^−1^ was determined using the PeroxiDetect Kit (Sigma-Aldrich, Saint Louis, MO, United States) after the extraction of 2.5 g of frozen peel in 10 ml of 5% trichloroacetic acid (TCA) based on the protocol described elsewhere (Giné-Bordonaba et al., 2019).

Malondialdehyde (MDA) was analyzed as an index of lipid peroxidation using the thiobarbituric acid reactive substrates (TBARS) based on the protocol previously described (Martínez-Solano et al., 2005) using 0.5 g of frozen tissue mixed with 4 ml of 0.1% trichloroacetic acid (TCA) solution.

### Ascorbic acid and dehydroascorbic acid content

Based on previous protocols (Rassam and Laing, 2005), the extraction of ascorbic acid was carried out mixing 2.5 g of frozen peel tissue with 5 ml of metaphosphoric acid suspension (3% metaphosphoric acid, 8% acetic acid) and centrifuging at 24,000 × *g* for 22 min at 4°C. Quantification of ascorbic acid (AsA) and total ascorbic acid was performed as previously described (Fernández-Cancelo et al., 2021). Dehydroascorbic acid (DHA) was calculated by subtracting the ascorbic acid content from that of total ascorbic acid.

### Determination of phenolic compounds and the peel antioxidant capacity

Total phenolic compounds (TPC) and antioxidant capacity of the apple peel were determined using frozen tissue as previously described (Giné Bordonaba and Terry, 2008) by mixing 3 g of apple frozen peel tissue with 10 ml of 79.5% (v/v) methanol and 0.5% (v/v) HCl in Milli-Q water. Sample extraction was held at 20°C with constant shaking for 2 h and mixing the samples every 30 min. The extract was centrifuged at 24,000 × *g* for 30 min at 20°C. From the same extract, total phenolic compounds were measured by means of the Folin–Ciocalteu method and total antioxidant capacity was measured by the Ferric Reducing Antioxidant Power (FRAP) assay as described in previous works (Giné-Bordonaba et al., 2016).

Individual phenolic compounds from peel tissue were quantified from the same extracts used for antioxidant capacity determination. The methanolic extract was filtered through a 0.22 μm filter and injected (5 μl) on an Agilent 1,260 Infinity II liquid chromatograph UHPLC (Agilent Technologies, Santa Clara, CA, United States) fitted with a Waters XSelect HSS T3 (4.6×100 mm, 2.5 μm; Waters Corporation, Milford, MA, United States) and a guard column Waters XSelect HSS T3 VanGuard (3.9×5 μm, 3.5 μm; Waters Corporation, Milford, MA, United States). Separation was carried out at a flow rate of 0.75 ml/min using a mobile phase consisting of 0.1% acetic acid in water (A) and 0.1% acetic acid in acetonitrile (B). The gradient program was held for the first 2 min at 6% B, increased linearly to 12% B in 3 min, then raised to 30% B in 29 min, and finally elevated to 100% B in 6 min and maintained at 100% B for 5 min. The temperature of the column was kept at 40°C and the sample compartment was refrigerated at 10°C. Detection was performed at 280, 320, and 520 nm, yet the online spectra were acquired in the 220–650 nm wavelength range with a resolution of 0.5 nm. Phenolic compounds were identified according to their retention time, spectral features, and ratios of maximum absorption peaks (λ). When no commercial standard was available, compounds were tentatively identified in an Acquity UPLC system equipped with diode array (DAD) and triple quadrupole mass spectrometer (MS) detectors using electrospray ionization interface (Waters, Milford, MA, USA). MS scan mode was used to obtain parent molecular ions and MS/MS daughters mode was used to obtain their fragmentation patterns, with argon as collision gas and 20–30 V as collision energies. Quantification was performed using calibration curves prepared with standard stock solutions of chlorogenic acid, catechin, and quercetin-3-O-glucoside, and their concentrations were expressed as g kg^−1^ FW. Detected phenolic acids and quercetin-derivatives were quantified using chlorogenic acid and quercetin-3-O-glucoside standard curves.

### Antioxidant enzymes activity

Peroxidase (POX, EC 1.11.1.7) was extracted by mixing 5 g of frozen peel with 10 ml of phosphate buffer (100 mM, pH 6) with 0.5 mM cysteine and 0.5 g of PVPP and centrifuged at 20,000 *g* for 15 min at 4°C. A 2.5 ml of supernatant was loaded into a Sephadex G-25 column (PD 10; Pharmacia, Madrid, Spain) that had previously been equilibrated with 10 ml phosphate buffer (100 mM, pH 6), and the enzyme was eluted with 3.5 ml of the same buffer. POX activity was determined as described elsewhere (Giné-Bordonaba et al., 2017).

Ascorbate peroxidase (APX; EC 1.11.1.11) extraction, 5 g of frozen peel was mixed with 15 ml of 100 mM base phosphate buffer (pH 7.5) containing 0.8 mM ascorbic acid and 1 mM EDTA. The mixture was centrifuged at 10000 *g* for 15 min at 4°C (Collazo et al., 2018). APX activity was determined at 290 nm for 10 min by monitoring the H_2_O_2_-dependent decomposition of ascorbate in a mixture containing 20 μl of supernatant and 280 μl of a reaction solution containing 0.22 mM ascorbic acid, 1 mM EDTA, and 1 mM H_2_O_2_ (Nakano and Asada, 1981).

Extraction of superoxide dismutase (SOD, EC 1.15.1.1) and catalase (CAT, EC 1.11.1.6) was carried out at the same time by homogenizing 5 g of peel tissue in 15 ml potassium phosphate buffer (100 mM, pH 7.8) containing 2 mM DTT, 5% (w/v) PVPP, 0.1 mM EDTA, and 1.25 mM polyethylene glycol (Chiriboga et al., 2013). The homogenate was centrifuged at 20000 *g* for 15 min at 4°C and then a 2.5 ml aliquot was loaded into a Sephadex G-25 column (PD 10; Pharmacia, Madrid, Spain) equilibrated with 10 ml 100 mM phosphate buffer (pH 7.8). The enzyme was eluted with 3.5 ml of the same buffer. The resulting supernatant was used as an enzyme extract to determine SOD and CAT activity. SOD activity was assayed by measuring its ability to inhibit the photochemical reduction of nitroblue tetrazolium (NBT) using the method described elsewhere (Giannopolitis and Ries, 1977). One unit of SOD (Unit of activity; UA) was considered the amount of enzyme required to inhibit NBT reduction by 50%. CAT activity was measured by the Claiborne method (Claiborne, 1986) following the disappearance of H_2_O_2_ at 240 nm. Except for SOD, enzymatic activity was expressed in UA per milligram of protein, with one UA representing the quantity of enzyme responsible for a change in 1 absorbance unit per minute. Protein was quantified by the method of Bradford (Bradford, 1976).

### Statistical data analysis

Data were subjected to analysis of variance (ANOVA) tests using JMP 13.1.0 SAS Institute. When the analysis was statistically significant, Tukey’s HSD test at the level *p* ≤ 0.05 was performed for comparison of means for the interaction location and development stage. A principal component analysis (PCA) was also performed to characterize the samples harvested at CHD in the four orchards studied. Data were centered and weighted using the inverse of the standard deviation of each variable in order to avoid the influence of the different scales used for the variables. Spearman’s rank correlation matrix (*p* ≤ 0.05) was done using the R corplot package.

## Results

### Orchard location influences peel color and composition

**Figure 1:**
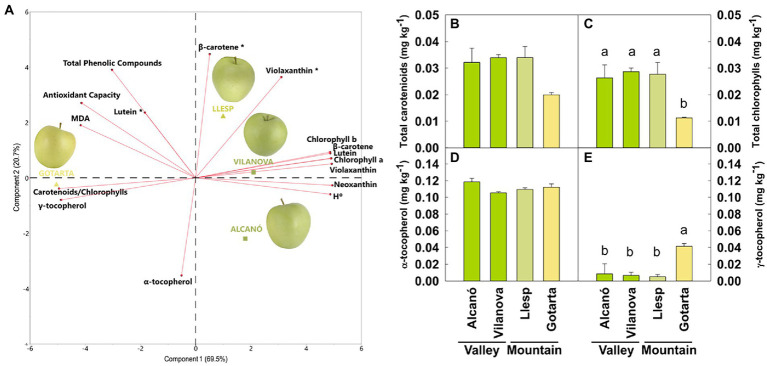
Principal component analysis (PCA) characterization of the apple peel from four different locations (two orchards from the valley: Alcanó and Vilanova; and two orchards from the mountain: Llesp and Gotarta) using 16 variables including Hue angle (H°) individual pigments and tocopherols, antioxidant capacity, TPC and MDA **(A)**. Total carotenoids **(B)**, total chlorophylls **(C)**, α-tocopherol **(D)**, and γ-tocopherol **(E)** in the peel of apples grown in different locations. Error bars represent the standard error (*n* = 4). Letters, whenever available, indicate significant differences between locations.

“Golden Reinders” apples grown in four different locations (two valleys – Alcanó and Vilanova and two mountains - Llesp and Gotarta - orchards) and picked at the CHD showed significant differences in peel color (determined both visually and as changes in Hue angle, H°) among locations, but only apples harvested in Gotarta mountain orchard exhibited an optimal yellow peel color (H° = 96 ± 0.8) if compared with fruit from valley locations—Alcanó (H° = 107 ± 0.5) and Vilanova (H° = 108 ± 0.4)—and even with apples harvested in the mountain orchard of Llesp (H° = 104 ± 0.7). A principal component analysis (PCA; Figure 1A) using individual isoprenoids (chlorophylls, carotenoids, and tocopherols), H°, MDA, TPC, and antioxidant capacity as variables was used to differentiate the apple peel from fruit grown at the four different locations (two valley vs. two mountain orchards). The first two principal components captured 90.2% of the total variability and were sufficient to clearly differentiate the samples. The bidimensional plot illustrated that greenish peel apples were clustered together in the PC1-positive part of the plot, while yellow apples were plotted in the negative part. Enhanced yellow coloration of Gotarta apples was accompanied by a distinct pigment and antioxidant profile as depicted in Figure 1A. Interestingly, no statistically significant differences on carotenoid content were found between the four orchards studied, being its levels ca. 0.03 ± 0.003 mg kg^−1^ DW (Figure 1B). However, chlorophyll levels on apples from Gotarta were around 2.5-fold lower if compared to fruit from the other orchards (Figure 1C), hence owning a higher carotenoid/chlorophyll ratio (ca. 1.8 in Gotarta vs. 1.2 in the green apples). Gotarta apples also presented higher levels of oxidative stress markers such as MDA (20 ± 1.7 μmol kg^−1^) if compared with valley apples (17 ± 2.3 μmol kg^−1^ and 16 ± 1.0 μmol kg^−1^ for Alcanó and Vilanova apples, respectively) and higher content of antioxidants, specifically γ-tocopherol (Figures 1D,E).

### Transcriptome reprograming in response to orchard location

**Figure 2:**
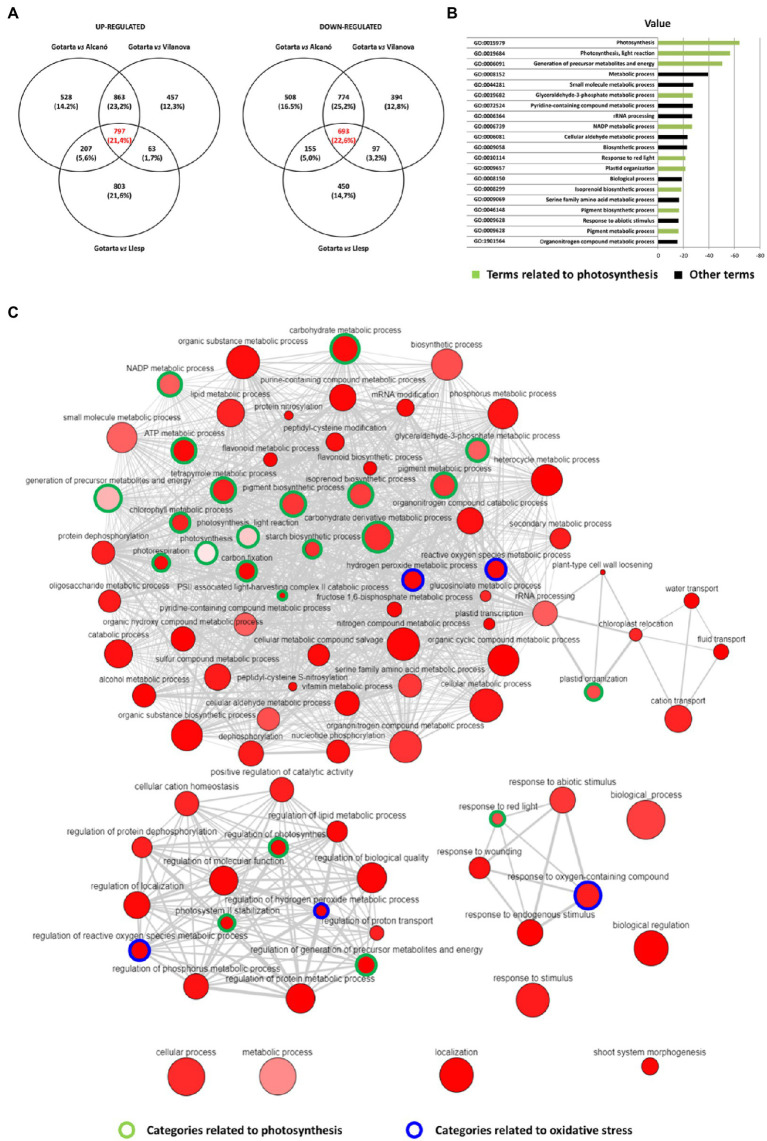
Number of genes upregulated and down-regulated **(A)** in Gotarta apples (yellow phenotype) compared with the other 3 orchards (green phenotypes). The 20 most frequent GO upregulated in Gotarta were displayed as % Total GO counted **(B)**. Gene functional categories enriched in Gotarta apples **(C)** provided by REVIGO’s “Scatterplot view” (http://revigo.irb.hr/). Disc color indicates enrichment value of p (the softer the color the highest the value) and size represents the frequency of the category. Thickness of grey lines represent semantic similarity between categories while spatial arrangement of discs corresponds to grouping of categories by semantic similarity. Those related to photosynthesis are circled in green, and terms associated with oxidative stress are circled in blue.

To study whether the expression of genes for enzymes in metabolic pathways involved in plastidial isoprenoid (chlorophyll, carotenoid, and tocopherol) biosynthesis and degradation was altered by the orchard location, a RNA-seq analysis of the peel from apples grown at the four different locations was performed (Figure 2; Supplementary Figure S1). Comparison of Gotarta (yellow phenotype) with the other three locations (green phenotype) showed that most genes of the carotenoid biosynthetic pathway were downregulated in Gotarta, whereas those involved in carotenoid degradation were upregulated, consistent with the observed trend toward lower levels of carotenoids in Gotarta apples (Supplementary Figure S1). By contrast, gene-encoding enzymes involved in chlorophyll degradation and tocopherol biosynthesis were strongly upregulated in “Gotarta” apples (Supplementary Figure S1).

To further identify other potential processes associated to color differences among orchards, a more detailed analysis of the RNA-seq data was performed (Figure 2). Comparison of Gotarta with the other three locations identified a common set of 797 genes that were upregulated in Gotarta and 693 genes that were downregulated if compared to all three locations leading to green apples (Figure 2A). GO term analysis of the whole set of differentially expressed genes (1,490 genes) showed an enrichment in processes related to photosynthesis, metabolism (including pigment and isoprenoid biosynthesis), plastid organization, and response to environmental cues (Figures 2B,C). Many terms within metabolism and cellular responses were actually related to photosynthesis, carbon fixation, and carbohydrate metabolic processes (Figure 2C), whereas another important group of GO terms was linked to response to oxidative stress, including genes involved in the regulation of ROS and H_2_O_2_ metabolism (Figure 2C). We also observed that pathways regulating nitrogen and phosphorus metabolic process as well as general organic substance metabolism were overrepresented among the set of differentially expressed genes. In terms of cellular responses, the regulation of biological quality, protein metabolic process, and molecular function was the most represented (Figure 2C).

### Pigment metabolism during fruit development is differentially affected by the environment

**Figure 3:**
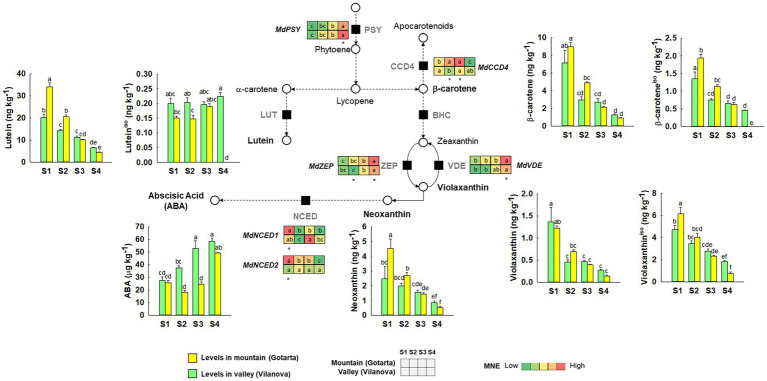
Comparison of carotenoid metabolism in “Golden Reinders” apples grown in mountain (Gotarta-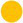) or valley (Vilanova-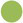) orchards during fruit development/ripening. Dashed lines indicate that some steps have been omitted. Quantified carotenoid compounds are represented in bar plots, including minor carotenoid isomers marked with the superscript “iso.” Error bars represent the standard error (*n* = 4). Letters, whenever available, indicate significant differences for the interaction location*developmental stage according to Tukey test (*p* ≤ 0.05). The corresponding transcript levels of the main genes are represented as heatmaps. Letters within the heatmaps indicate significant differences (*p* ≤ 0.05) between development stages, while asterisk denote differences among locations at each specific development stage.

Based on the major differences on peel color, pigment composition, and gene expression, Gotarta (mountain) and Vilanova (valley) orchards were selected to further monitor major metabolic and transcriptomic shifts associated to fruit color changes along fruit development (four developmental stages: S1 to S4, the latter being the CHD). Differences in carotenoid levels were observed between locations and developmental stages, yet the carotenoid profile, understood as the individual carotenoids identified among samples, remains relatively unchanged (Figure 3). Lutein was the main carotenoid found in the apple peel, accounting for 60% of total carotenoids. β-carotene and violaxanthin each contributed to 15% of the total carotenoids blend, while neoxanthin accounted only for the 8% of total carotenoids. In both locations, carotenoids content decreased as the fruit ripened on-tree, yet the reduction rate was 2-fold higher in mountain than in valley fruit. In consequence, although carotenoid levels were *ca.* 30% higher in Gotarta at the two initial samplings (S1 and S2), carotenoid content in mountain apples reached similar levels to those observed in valley apples in the last two samplings (S3 and S4) (Supplementary Figure S2A). The global decrease in carotenoid content during fruit development in both locations only translated into an increase of ABA from 27 ± 2.9 to 59 ± 2.5 μg kg^−1^ in valley fruit from S1 to S3 (Figure 3). In contrast, ABA levels in mountain fruit remained around 23 ± 2.2 μg kg^−1^ DW until S4, when it reached similar levels to those found in valley apples (49 ± 0.4 μg kg^−1^ DW). Chlorophyll levels followed the same pattern as carotenoids in both locations (Supplementary Figure S2B). Mountain apples accumulated a 40% more chlorophyll in the first two stages of development (S1 and S2) and then its levels matched with those from the valley at S3 and S4 (0.02 ± 0.001 and 0.01 ± 0.001 mg kg^−1^ DW, respectively).

Carotenogenic gene expression (*MdPSY*, *MdVDE,* and *MdZEP*) progressively increased during fruit development for both studied locations (Supplementary Figures S3A–C). In the last stage, *MdPSY* expression was 1.4-fold higher in Vilanova whereas *MdZEP* and *MdVDE* showed a 30% greater expression in Gotarta. Regarding gene-encoding apocarotenoid production, *MdCCD4* expression remained constant along fruit development in the valley, while it increased from S1 to S2 and significantly declined at S4 in mountain apples (Supplementary Figure S3D). The expression of *MdNCED1* and *MdNCED2* was similar between locations and remained unchanged during fruit development except at the first stage (S1), where mountain apples showed 3-fold higher expression of both genes in comparison to fruit from the valley (Supplementary Figures S3E,F).

### The levels of tocopherols and phenolic compounds are greater in mountain apples

Mountain apples showed 10-fold higher levels of γ-tocopherol at S4 if compared with valley apples (Supplementary Figure S2C), whereas during earlier stages (S1–S3), γ-tocopherol content remained below 0.004 mg kg^−1^ DW and constant in both locations. In contrast, α-tocopherol remained unchanged (0.03 ± 0.003 mg kg^−1^ DW) in both locations during fruit development and ripening on-tree (Supplementary Figure S2D).

**Figure 4:**
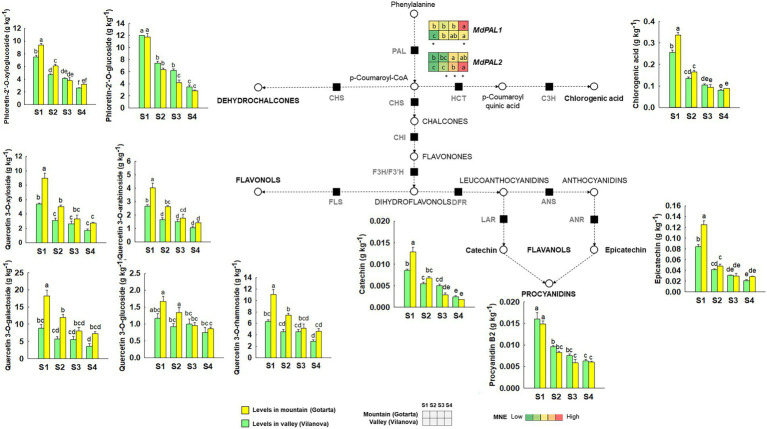
Comparison of phenolic compounds metabolism in “Golden Reinders” apples grown in (Gotarta-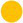) or valley (Vilanova-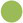) orchards during fruit development/ripening. Dashed lines indicate that some steps have been omitted. Phenolic compounds are represented in plots. Error bars represent the standard error (*n* = 4). Letters, whenever available, indicate significant differences for the interaction location*developmental stage according to Tukey’s test (*p* ≤ 0.05). The corresponding transcript levels of the main genes are represented as heatmaps. Letters within the heatmaps indicate significant differences (*p* ≤ 0.05) between development stages, while asterisk denote differences among locations at each specific development stage.

Eleven phenolic compounds were identified in both locations during fruit development, including phenolic acids (chlorogenic acid), dihydrochalcones (phloretin glycosides), flavanols (catechin, epicatechin, and procyanidin B2), and flavonols (quercetin glycosides; Figure 4). In general, the peel from mountain apples showed higher levels of phenolic compounds in the two first development stages (S1 and S2), except for phloretin-2’-O-glucoside and procyanidin-B2 whose levels were not affected by the orchard location. All phenolic compounds identified followed a downward trend in both location as the fruit developed, being the decrease rate between 1.2 and 2.0-fold higher in mountains than in valley apples yet depending on the phenolic compound (Figure 4). At the CHD, despite most differences between locations levelled off, the peel from mountain apples showed generally higher levels of phenolic compounds than those from the valley.

Analysis of *MdPAL1* and *MdPAL2* showed expression differences between locations mainly at S4, being significantly higher and lower the expression of *MdPAL1* and *MdPAL2* in mountain apples than in fruit from the valley.

### The environment strongly influences ROS-scavenging enzymes in the apple peel

**Figure 5:**
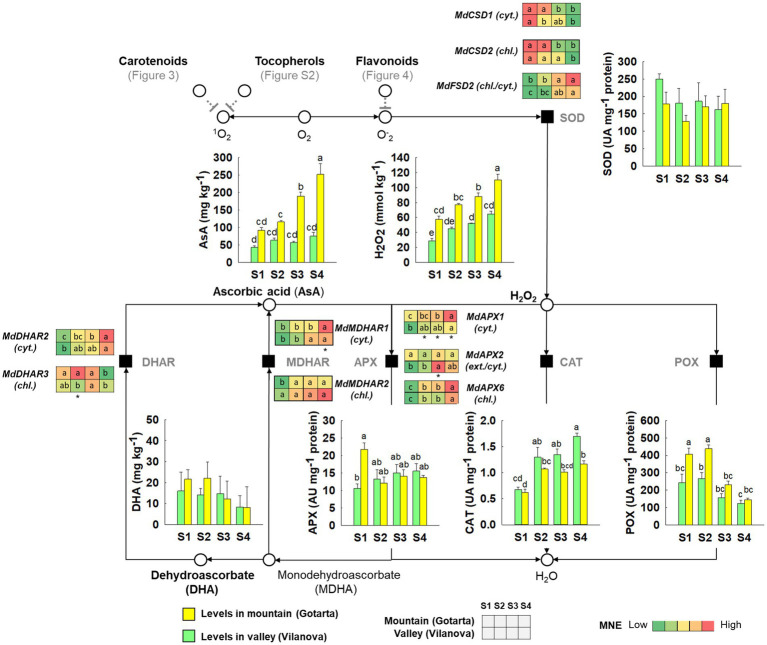
Comparative overview of the ROS-scavenging metabolism and ascorbate recycling pathway of “Golden Reinders” apples grown in mountain (Gotarta-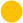) or valley (Vilanova-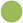) orchards during fruit development/ripening. The activity of the main antioxidant enzymes, as well as the content of ascorbic acid and H_2_O_2_, is represented in bar plots. Error bars represent the standard error (*n* = 4). Letters, whenever available, indicate significant differences for the interaction location*stage according to Tukey test (*p* ≤ 0.05). The corresponding transcript levels of the main genes are represented as heatmaps. Letters indicate significant differences (*p* ≤ 0.05) between development stages, while asterisk denote differences among locations at each specific development stage.

A completely different pattern of H_2_O_2_ accumulation was observed when comparing apples grown in mountain or valley orchards. Levels of H_2_O_2_ remained unchanged in valley apples from S2 to S4 (around 54 ± 4.2 mmol kg^−1^), whereas in mountain apples, H_2_O_2_ raised from 77 ± 2.0 to 110 ± 8.1 mmol kg^−1^ within the same period (Figure 5). Enhanced H_2_O_2_ accumulation in the peel from mountain apples was not associated to higher levels of SOD, the enzyme responsible for the dismutation of O_2_^·-^ into H_2_O_2_, since the activity of this enzyme remained relatively unchanged throughout fruit development/ripening in both locations. In contrast, the activities of enzymes involved in the decomposition of H_2_O_2_ into water and oxygen were location and developmental stage dependent. The activity of APX, the enzyme responsible to catalyze the H_2_O_2_-dependent oxidation of ascorbate was nearly 2-fold greater in mountain apples at S1, but it decreased in the rest of developmental stages to 13 ± 1.5 UA mg^−1^ protein, matching then the activity levels found in valley apples. Ascorbic acid levels in the valley remained unchanged with values below 75 mg kg^−1^, whereas AsA levels massively increased from 92 ± 8.0 to 252 ± 30.0 mg kg^−1^ in mountain apple peel during fruit development and on-tree ripening. A similar pattern was observed for POX, whose activity was significantly higher in mountain apples at S1 and S2 (Figure 5). In the last two samplings (S3 and S4), POX activity from mountain apples decreased and matched the levels observed in fruit from the valley (134 ± 4.5 UA mg^−1^ protein). In contrast, no differences in CAT activity were observed between valley and mountain apples except at S4, when CAT activity in fruit from the valley increased until reaching values of 1.7 ± 0.07 UA mg^−1^ protein. Significant differences were found for the expression of all genes encoding for antioxidant enzymes along the different fruit development stages and locations except for *MdDHAR3* and *MdMDHAR2* that showed no changing pattern along fruit development/ripening (Supplementary Figure S5). Copper superoxide dismutase encoding genes (*MdCSD1* and *MdCSD2*) showed the same downward pattern in both locations along fruit development/ripening. However, the expression of *MdCSD2*, which encodes for chloroplastic SOD, was around 40-fold higher than its cytosolic counterpart *MdCSD1* (Supplementary Figures S5A,B). In turn, *MdFSD2* showed the opposite trend, reaching maximum expression levels at S4 for both locations (Supplementary Figure S5C). In the case of *MdAPX1,* encoding for cytosolic APX, only mountain apples showed an increase of gene expression from 30 to 60 MNE during development/ripening (Supplementary Figure S5D). The expression of *MdAPX6* and *MdMDHAR*1 increased during fruit development/ripening and significant differences were found for the expression of these genes at the CHD (Supplementary Figures S5F,G).

## Discussion

### Optimal yellow peel color in “Golden Reinders” apples is associated to oxidative stress

“Golden Reinders” apples were grown under similar agronomical practices in four orchards of Lleida Province (two from the valley and two from the mountains) and harvested at the same ripening status in terms of fruit size (75 ± 0.4 mm), firmness (71 ± 0.5 N) and other ripening-related traits (Fernández-Cancelo et al., 2021) showed different peel color phenotype (Figure 1). Notably, fruit from mountain orchards, and especially those from Gotarta, showed enhanced yellow color, as desired by consumers, but also higher antioxidant capacity and elevated levels of MDA, a marker of lipid peroxidation (Hodges et al., 1999). Since the apples were harvested at a similar maturity stage (Fernández-Cancelo et al., 2021), climatic differences between orchards may have influenced peel metabolism (McTavish et al., 2020) leading to different color phenotypes. Indeed, in our previous study, we demonstrated that optimal fruit color in “‘Golden Reinders” apples was not associated to the fruit maturity stage and that fruit grown in the Ebro valley never attain the optimal yellow color desired by the consumer, even if left to ripen on-tree far beyond the CHD (Fernández-Cancelo et al., 2021). Among the wide variety of environmental factors that can influence fruit color (temperature, solar radiation, hours of sun, precipitations, and relative humidity), the low temperatures and solar radiation reached days before harvest in Gotarta (Supplementary Figures S8, S9) in combination with a probable higher accumulation of UV radiation due to the high altitude (data not measured; Blumthaler et al., 1997) may have favored chlorophyll degradation (León-Chan et al., 2017) and color changes in Gotarta apples.

Given the difficulties to evaluate the specific environmental factors affecting apple color, we decided to focus our study in investigating the metabolic or transcriptomic shifts capable to explain the color phenotype differences observed between growing locations, with a special emphasis on pigment and antioxidant metabolism. The pigment profiling presented herein revealed that yellow apples had similar levels of carotenoids but significant lower levels of chlorophylls than apples grown at other locations and showing greenish coloration (Figure 1). These observations strongly suggest that higher chlorophylls levels in greenish “Golden Reinders” apples may be shielding the carotenoid contribution to peel color as it was described in pepper (Filyushin et al., 2020). Hence, it may be that chlorophyll levels in apple peel must fall below a certain threshold for achieving an optimal yellow color as previously observed during olive ripening (Mínguez-Mosquera and Gallardo-Guerrero, 1995). Besides, our data demonstrated that enhanced yellow color was not due to enhanced content of carotenoids, as it may be deduced from available literature (Delgado-Pelayo et al., 2014) but rather associated to higher carotenoids/chlorophyll ratios triggered by environmental cues. Yellow peel apples showed also significantly higher levels of tocopherols which are antioxidant molecules involved in the protection of thylakoid membranes against lipid peroxidation (Piller et al., 2014). These observations, together with the results of the RNA-seq analyses showing an overrepresentation of gene categories related to photosynthesis and oxidative stress responses (Figure 2), strongly suggest that the yellow peel of Gotarta apples may be the result of higher exposure to oxidative damage.

### Pigments and phenolic compounds decrease during fruit development and ripening

Based on the intense color differences observed between Vilanova and Gotarta apples, both locations were chosen as the most suitable to better understand the metabolic and transcriptomic differences underlying different apple phenotypes. To elucidate the point when color changes and pigment differences occur, apple development was followed at four different stages (S1 to S4) based on fruit size in both valley (Vilanova) and mountain (Gotarta) orchards. The optimal yellow color was only perceived in Gotarta apples at the last developmental stage (S4; equivalent to the CHD), while in Vilanova, the apple peel remained green (Table 1). Color changes in fruit are generally associated to the transition from chloroplast to the chromoplast and the accumulation of carotenoids (Sadali et al., 2019). However, the observed differences in “Golden Reinders” peel color were again not due to enhanced accumulation of carotenoids but rather to a more intense degradation of chlorophylls (Supplementary Figure S2A). The characteristic decrease of chlorophyll and carotenoids in yellow peel apples (Solovchenko et al., 2005) was observed in both locations, yet the final carotenoid/chlorophyll ratio was higher in the mountain (Gotarta) location (Table 1). This accelerated degradation of chlorophylls not only explains the more yellowish color of these apples but also the increased levels of tocopherols (Figure 1), which in many fruits are made from phytol recycled from the chlorophylls degraded during ripening (Muñoz and Munné-Bosch, 2019). Differences in the chlorophyll and tocopherol levels may be driven, in turn, by differences in temperature and solar radiation among the studied locations (Karagiannis et al., 2020). In fact, a higher exposure to sunlight is known to favor the accumulation of chlorophyll and carotenoids in apple (Jing et al., 2020), while an excess of it can also induce apple ripening and accelerate chlorophyll loss (Solovchenko et al., 2006). Higher solar radiation combined with milder temperatures during the early development stages may have favored the accumulation of photosynthetic pigments (chlorophylls and carotenoids) in mountain locations at S1 and S2 (Supplementary Figures S2, S8, S9). As summer progressed, the pronounced decrease in solar radiation and temperatures in mountains possibly favored, to some extent, the inhibition of pigment biosynthesis (Nagata et al., 2007) and, hence, a more pronounced decline in the chlorophyll and carotenoids content in mountain apples if compared with those from the valley (Supplementary Figures S2, S8, S9).

**Table 1:** Peel color evolution during the four developmental stages studied in Vilanova (valley) and Gotarta (mountain) orchards.

	S1 (60% final fruit size)	S2 (80% final fruit size)	S3 (90% final fruit size)	S4 (100%final fruit size)
**Vilanova** (Valley Orchard)	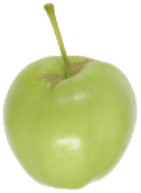	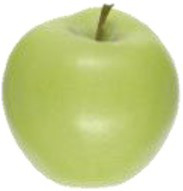	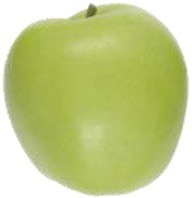	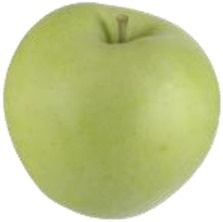
H^o^	104.5 ± 0.14 ab	104.2 ± 0.16 bc	103.6 ± 0.27 bc	100.5 ± 0.32 e
Carotenoids/Chlorophylls	1.05 ± 0.023 a	0.95 ± 0.024 bc	0.98 ± 0.021 abc	0.94 ± 0.016 c
**Gotarta** (Mountain Orchard)	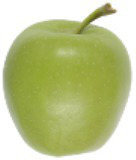	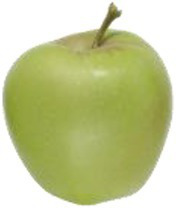	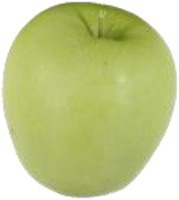	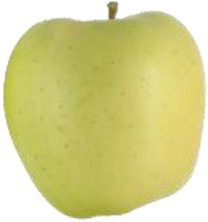
H^o^	105.1 ± 0.12 a	103.3 ± 0.21 c	102.2 ± 0.24 d	95.8 ± 0.38 f
Carotenoids/Chlorophylls	1.03 ± 0.005 ab	1.03 ± 0.017 abc	0.96 ± 0.027 abc	1.03 ± 0.018 abc

Hue angles (H°) were determined by measuring both sides of 30 fruits (*n* = 60), while the ratio carotenoids/chlorophylls were determined from four replicates (*n* = 4). Data are represented as the means of replicates ± standard error. Letters indicate significant differences for the interaction location*developmental stage according to Tukey’s test (*p* ≤ 0.05).

The differences in the temporal changes of carotenoids along fruit development between locations were not a consequence of differences on the expression of *MdPSY*, the main regulator of carotenoid synthesis (Cazzonelli and Pogson, 2010). The upward trend in *MdPSY* expression in both locations did not lead to an accumulation of carotenoids, suggesting the possibility that degradation pathways were more active and rather responsible for the final levels of carotenoids observed in apples at the time of harvest (Figure 3). Nonetheless, carotenoid catabolism seemed to be strongly influenced by the orchard location. The upregulation of *MdCCD4* in S2 and S3 combined with the absence of correlation between carotenoids and ABA observed in mountain apples, suggest that high-altitude environments may favor alternative degradation pathways than those mediated by NCED, hence in agreement with available literature for other organisms (Liang et al., 2021). Indeed, the accumulation of ROS in mountain apples (Figure 5) may favor the synthesis of stress response apocarotenoids through the CCD4 pathway (Felemban et al., 2019). The pattern of ABA accumulation may be, in turn, influenced by temperature differences between locations since ABA is considered a stress response hormone involved in thermotolerance (Iqbal et al., 2021). In this regard, apple is a crop adapted to moderate climates (Funes et al., 2016); hence, the high temperatures reached in Vilanova during fruit development may activate the accumulation of ABA for better coping with heat stress (Torres et al., 2017). Likewise, the moderate temperatures in mountain during the three first stages (Supplementary Figure S8) likely kept ABA levels low until the last stage, when ABA may be induced not by warm but rather by cold temperatures (Lee et al., 2021). Indeed, evidence suggesting that ABA accumulates in response to high- or low-temperature stress is abundant for other species (Daie and Campbell, 1981), yet no data are currently available for apples. Future studies are encouraged to better understand the actual relationship between ABA content and environmental temperatures during apple growth.

Besides carotenoids, tocopherols play an important role in maintaining chloroplast integrity. In our study, α-tocopherol levels in the apple peel remained stable during fruit ripening in both locations, whereas a burst in γ-tocopherol was observed in mountain apples at the latest stage (S4). Given that tocopherol synthesis utilizes phytyl-PP released during chlorophyll breakdown, it is plausible to hypothesize that the drop of night temperatures observed in high-altitude orchards days before harvest (Supplementary Figure S8) may strongly activate phytol recycling mechanisms and promote the accumulation of γ-tocopherol (Vom Dorp et al., 2015), thereby enhancing stress tolerance (Abbasi et al., 2007). The decrease in chlorophyll content and the accumulation of oxidative stress markers observed in Gotarta apples (Figures 3, 5) are signs compatible with senescence (Muñoz and Munné-Bosch, 2018). Senescence can be activated by environmental cues such as the low temperatures and low solar radiation reached in Gotarta just before harvest (Supplementary Figures S8, S9), or also by an accumulation of ROS (Figure 5) able to cause a potential oxidative damage in the peel of Gotarta apples (Thakur et al., 2016). Hence, the development of yellow color in mountain apples may be interpreted as the result of an activation of a peel senescence-like process driven by environmental stresses. However, the RNA-seq analysis did not show a differential expression of senescence-associated genes (SAGs) between locations able to support this hypothesis, and therefore further studies are warranted.

Although carotenoids and tocopherols play a key role as antioxidants in chloroplasts, the total antioxidant capacity in apple mainly depends on phenolic compounds (Lee et al., 2003). Phenolic compounds can be subdivided into different classes including hydroxycinnamic acid derivatives, chalcones, flavanols, flavonols, and anthocyanins (Haminiuk et al., 2012). The main phenolic compounds detected in “Golden Reinders” peel were chlorogenic acid and quercetin-3-O-glycosides (Figure 4), which followed the same trend as carotenoids, decreasing as the fruit developed and ripened, in accordance with previous studies made on apple (Wojdyło and Oszmiański, 2020). The higher levels of flavanols, flavonols, and hydroxycinnamic acid derivatives found at early development stages in mountain fruit may also be a consequence of the higher solar radiation received by apple peel at high altitudes in the first months of fruit growth (Awad et al., 2000).

The first step of the phenolic compound synthesis pathway is catalyzed by Phenylalanine Ammonia-Lyase (PAL), an enzyme encoded by several gene isoforms regulated by the environment (Huang et al., 2010). Extensive evidence has shown that PAL gene expression in model species (i.e., *Arabidopsis thaliana*) is responsive to a variety of environmental stimuli, including pathogen infection, wounding, nutrient depletion, UV irradiation, and extreme temperatures, among others (Huang et al., 2010). Accordingly, the PAL gene isoforms analyzed in our study (*MdPAL1* and *MdPAL2*) showed different expression patterns (Supplementary Figure S4) not only along fruit growth but also in response to the growing location, suggesting that each isoform may be differentially regulated by environmental stimuli. In this sense, the observed *MdPAL1* gene expression pattern and the high correlation with γ-tocopherol (Supplementary Figure S7) suggest that both *MdPAL1* gene expression and γ-tocopherol may be regulated by similar environmental cues, being in this particular case, likely low temperatures prior to the time of harvest (Maeda et al., 2006; Olsen et al., 2008). *MdPAL2* gene expression, in contrast, seemed to mainly change along fruit developmental, showing an upward trend as the fruit developed and ripened yet with more pronounced and significant higher expression levels in fruit grown in the valley at S4. In this particular case, and based on our metereological data, enhanced UV or solar radiation at this particular phenological stage may account for the higher *MdPAL2* gene expression in valley fruit. Overall, our results are in agreement with previous studies showing a differential expression of *PbPAL* genes among different tissues, phenological stages or even in response to exogenous hormonal treatments in other pome fruit species (i.e., pears; Li et al., 2019). Nonetheless, further studies are encouraged to elucidate the possible specialization or environmentally driven regulation of *MdPAL* genes in apple fruit.

### The accumulation of H_2_O_2_ favored by the mountain environment may trigger apple peel degreening

In addition to the antioxidant molecules described above, fruit has enzymatic mechanisms able to neutralize toxic ROS species generated by aerobic metabolism and stress (Apel and Hirt, 2004). The RNA-seq data showed that a large number of genes related to oxidative stress were differentially expressed when comparing yellow and green peel “Golden Reinders” apples (Figure 2C). In this sense, we further investigated changes in the activity of enzymes involved in ROS-homeostasis during the last stages of apple development/ripening in both locations (mountain vs. valley; Figure 5). The enzyme SOD is present in the cytoplasm, mitochondrion, and the chloroplast (Feng et al., 2016), and is able to convert oxygen radicals produced in the processes of photosynthesis and respiration into a less reactive compound such as H_2_O_2_ (Meitha et al., 2020), which can be reduced by peroxidases present in cytosol and chloroplast (Maruta et al., 2016) and by catalase in peroxisome (Corpas, 2015). Although orchard location and developmental stages influenced SOD gene expression levels, SOD activity remained unchanged regardless of the environment. In contrast, peroxidase activities tended to be higher in the peel of mountain apples for POX in S1 and S2 and for ascorbic acid-dependent peroxidase (APX) in S1 (Figure 5). In general, the expression of genes involved in ascorbate metabolism or the antioxidant enzymes detailed above was poorly correlated with the equivalent enzymatic activity but well correlated with the concentration of the corresponding metabolite (Supplementary Figure S7). Based on the absence of correlation between APX activity, ascorbic acid (AsA), and the gene expression of ascorbate recycling enzymes *MdMDHAR* and *MdDHAR* (Supplementary Figure S7), it is possible to speculate that the differential accumulation pattern of ascorbic acid found between locations may be a mere consequence of a higher biosynthetic activity driven by environmental cues (Zhang et al., 2011). Furthermore, the high correlation between AsA and H_2_O_2_ (Supplementary Figure S7) suggests that AsA may play an important role in modulating the protection against ROS (Akram et al., 2017). In this sense, the increase of H_2_O_2_ levels observed in the peel of mountain-grown apples may be favored by the reduction of peroxidase activities during fruit development combined with the low catalase activity (Figure 5). Since during the early development stages (90 days before harvest) climatic differences were not appreciable between valley and mountain orchards, we would like to point out that other environmental stresses such as higher UV radiation incidence on mountain apples may induce the accumulation of H_2_O_2_ (Nawkar et al., 2013). Given that fruit ripening is considered an oxidative process by itself (Steelheart et al., 2019), it is reasonable to hypothesize that the accumulation of H_2_O_2_ in mountain apples may trigger peel ripening associated processes such as color changes (Kumar et al., 2016) without affecting pulp ripening parameters such as firmness or starch index (Fernández-Cancelo et al., 2021). These results would further reinforce the results from recent studies suggesting that apple peel and flesh ripening are independent processes (Karagiannis et al., 2020).

The phenotypic differences observed between locations may be regulated epigenetically through DNA methylation and specific histone modifications driven by the environment (Baulcombe and Dean, 2014). Moreover, recent studies have shown that the DNA methylation can regulate pigment accumulation in apple (Jiang et al., 2019) but little is known about the involvement of histone modifications in apple color. Given that some deacetylases seem to have a key role in the modulation of color development in tomato (Guo et al., 2017) and since environmental stress may also alter the epigenetic regulation (Baulcombe and Dean, 2014), we decided to perform a gene expression analysis of a class of histone deacetylases known as sirtuins (*MdSRT1* and *MdSRT2*). Sirtuins are suggested to be involved in the epigenetic regulation of fruit ripening in other Rosaceae spp. (Vall-llaura et al., 2021) and well known for their association to several antioxidant and oxidative stress-related processes and functions (Singh et al., 2018). Indeed, the fact that sirtuins deacetylase activity is dependent on NAD^+^, a key redox signaling molecule, sustains the idea that these molecules may be integral players in regulating cellular antioxidant and redox signaling pathways (Singh et al., 2018). In our study, the high correlation between the expression of *MdSRT2* and ripening associated traits such as Hue angle (H°), H_2_O_2_, and AsA levels (Supplementary Figure S7) suggests that fruit ripening and color changes may be induced epigenetically through the action of histone deacetylases as described in tomato (Guo et al., 2017). On the other hand, the analysis of *MdSRT1* gene expression, a sirtuin involved in abiotic stress responses in *A. thaliana* (Liu et al., 2017), showed a consistently higher expression in mountain apples at all ripening stages (Supplementary Figure S6). Overall, the expression analysis of sirtuins may point out that mountain apples suffer an additional stress during fruit growth able to induce epigenetic changes that lead to an acceleration of ripening processes including color changes. However, further studies should be performed to analyze which specific epigenetic modifications regulate peel color in “Golden Reinders” apples.

## Conclusion

The results from this study demonstrate that differences in apple peel color among valley and mountain orchards at the time of harvest are explained by the influence of environmental conditions on antioxidant and pigment metabolism occurring earlier during fruit development. Optimal yellow coloration in “Golden Reinders” apples, as exclusively observed in mountain environments, was mainly a consequence of enhanced chlorophyll degradation instead of enhanced carotenoid levels as well as alterations in the expression of genes involved in chloroplasts functions. Likewise, the yellow phenotype observed in mountain apples seems also to be regulated through epigenetic modifications driven by the environment and may be related, to some extent, to oxidative stress and to the specific action of H_2_O_2_ during fruit growth/ripening_._ In this sense, H_2_O_2_ seemed to play a key role triggering some peel ripening events, including those involved in peel degreening in fruit grown in the mountains. Further studies may be required to dissect how specific environmental cues (solar radiation, temperature and so on) can trigger or inhibit pigment and antioxidant metabolism in different apple varieties hence leading to fruit with different phenotypes. Besides, the results from this study may assist in selecting specific compounds that when applied exogenously are capable to trigger color changes in fruit grown under specific environments.

## Data availability statement

RNA-seq raw data are freely available in SRA database with the Bioproject ID PRJNA839574. Other data supporting the findings of this study are available within the paper and within its Supplementary material published online.

## Author contributions

PF-C: methodology, writing—original draft preparation, and writing—review and editing. AI-S, AR-A and ST-M: methodology and writing—review and editing. NT: supervision and writing—review and editing. MR-C and JG-B: conceptualization, methodology, supervision, funding acquisition, project administration, and writing—review and editing. All authors contributed to the article and approved the submitted version.

## Funding

This work has been financially supported by the Spanish Agencia Estatal de Investigación (AEI) and European Regional Development Fund (ERDF) through the national projects RTA2015-00037-CO2-01, BIO2017-84041-P, BIO2017-90877-REDT and PID2020-115810GB-I00. Funding was also provided by the internal CRAG project “Green2Gold” in the context of the Severo Ochoa Programme for Centres of Excellence in R&D 2016–2019 (SEV-2015-0533). This work has been also supported by the CERCA Programme from the “Generalitat de Catalunya.” Thanks are also given to AEI and ERDF for the predoctoral fellowships awarded to PF-C (BES-2017-080741) and AI-S (PRE2018-083610). ST-M is supported by a PhD fellowship from the Spanish Ministry of Education, Culture and Sports (FPU16/04054).

## Conflict of interest

The authors declare that the research was conducted in the absence of any commercial or financial relationships that could be construed as a potential conflict of interest.

## Publisher’s note

All claims expressed in this article are solely those of the authors and do not necessarily represent those of their affiliated organizations, or those of the publisher, the editors and the reviewers. Any product that may be evaluated in this article, or claim that may be made by its manufacturer, is not guaranteed or endorsed by the publisher.

## Footnotes

^1^ https://www.transcriptomics.cloud

^2^ https://www.rosaceae.org/

^3^ https://www.rosaceae.org/

^4^ https://systemsbiology.cau.edu.cn/agriGOv2

^5^ https://revigo.irb.hr
