# Supplementary material for: Environmentally driven transcriptomic and metabolic changes leading to color differences in “Golden Reinders” apples
Source: Front Plant Sci. 2022 Aug 1;13:913433. doi: 10.3389/fpls.2022.913433 (PMC9377453; doi:10.3389/fpls.2022.913433)
Supplement: Supplementary file 1 [file Presentation_1.PPTX]

## Slide 1
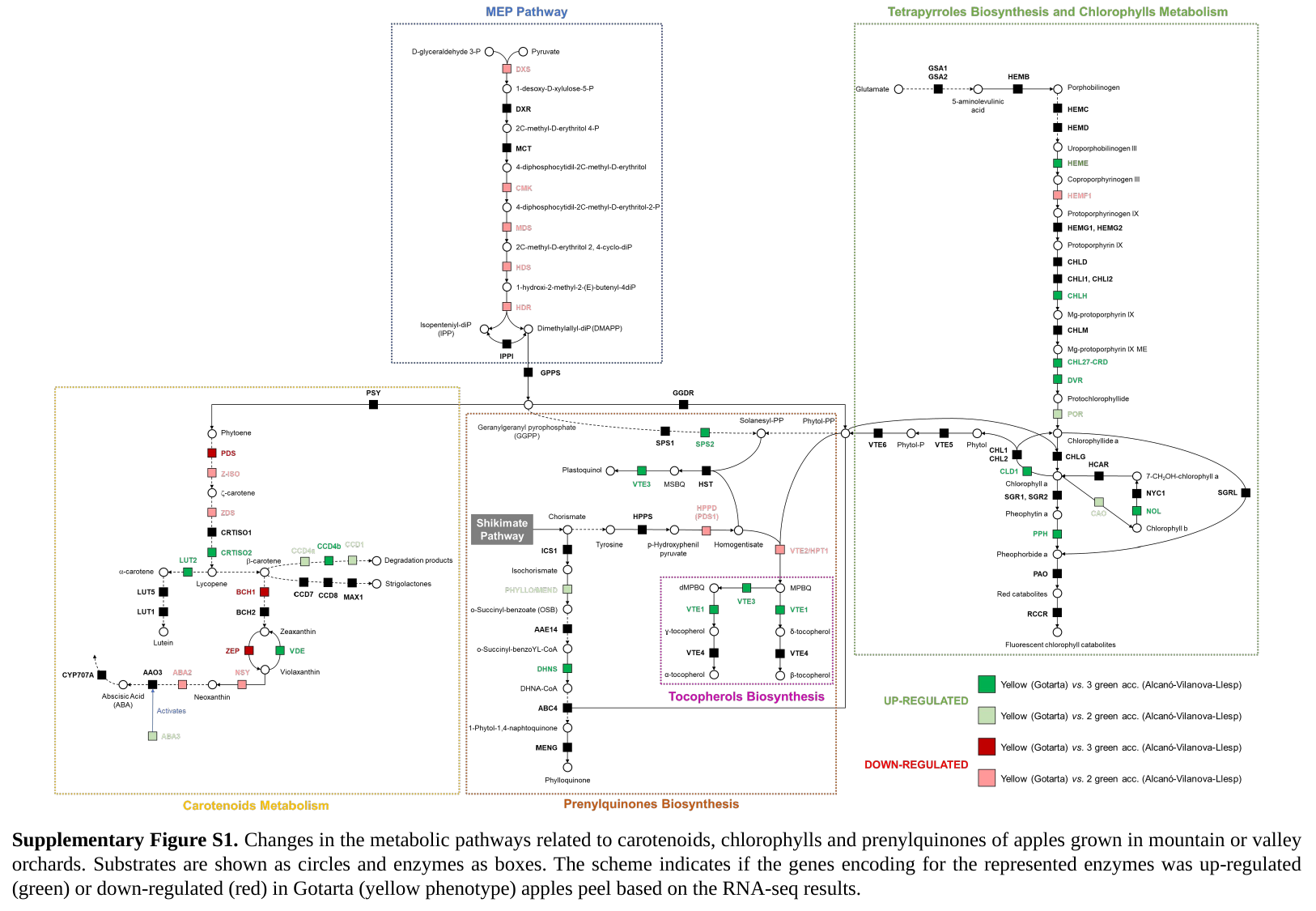

Supplementary Figure S1. Changes in the metabolic pathways related to carotenoids, chlorophylls and prenylquinones of apples grown in mountain or valley orchards. Substrates are shown as circles and enzymes as boxes. The scheme indicates if the genes encoding for the represented enzymes was up-regulated (green) or down-regulated (red) in Gotarta (yellow phenotype) apples peel based on the RNA-seq results.

## Slide 2
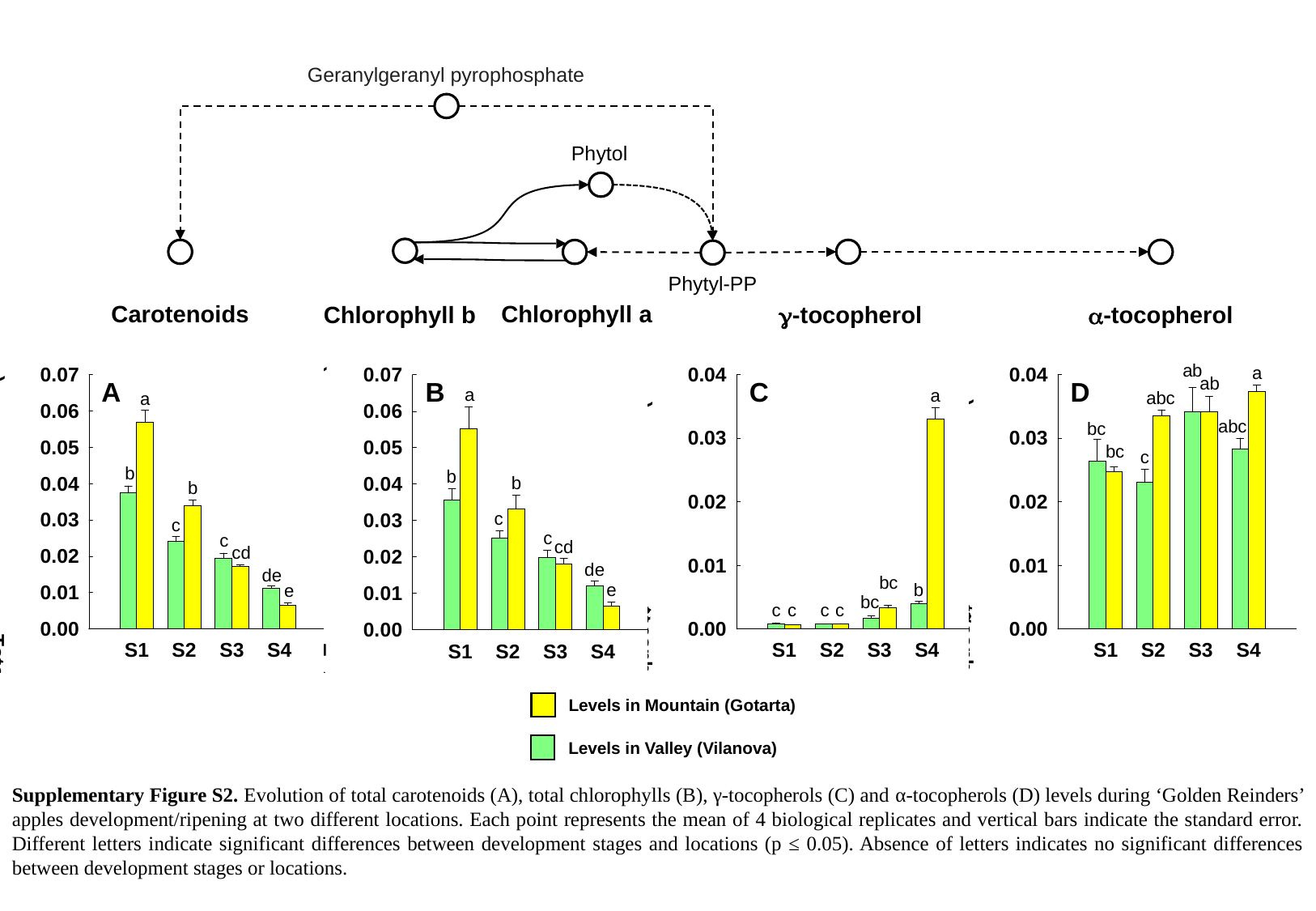

Geranylgeranyl pyrophosphate
Phytol
Phytyl-PP
Carotenoids
Chlorophyll a
Chlorophyll b
g-tocopherol
a-tocopherol
A
B
C
D
Levels in Mountain (Gotarta)
Levels in Valley (Vilanova)
Supplementary Figure S2. Evolution of total carotenoids (A), total chlorophylls (B), γ-tocopherols (C) and α-tocopherols (D) levels during ‘Golden Reinders’ apples development/ripening at two different locations. Each point represents the mean of 4 biological replicates and vertical bars indicate the standard error. Different letters indicate significant differences between development stages and locations (p ≤ 0.05). Absence of letters indicates no significant differences between development stages or locations.

## Slide 3
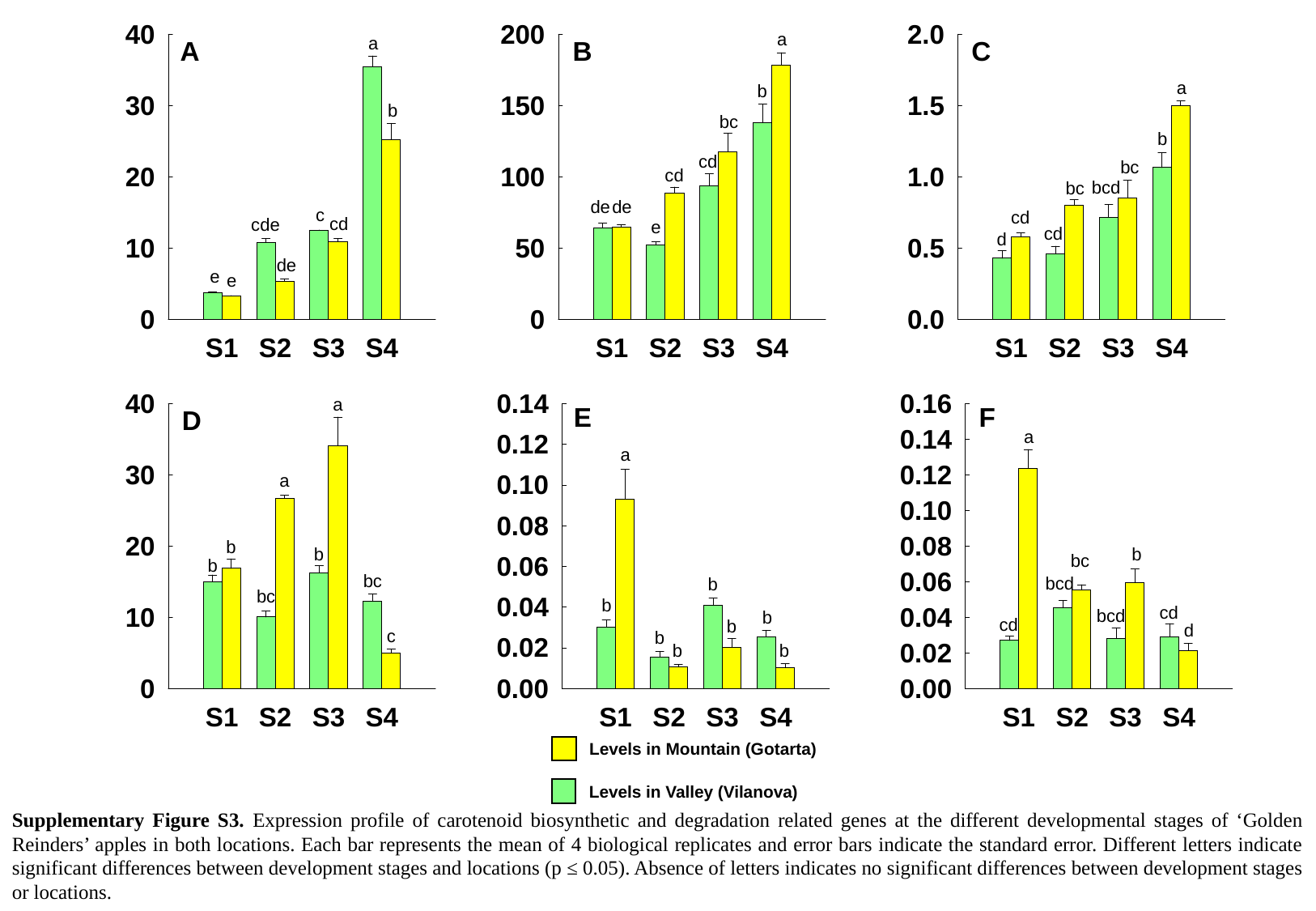

A
B
C
E
F
D
Levels in Mountain (Gotarta)
Levels in Valley (Vilanova)
Supplementary Figure S3. Expression profile of carotenoid biosynthetic and degradation related genes at the different developmental stages of ‘Golden Reinders’ apples in both locations. Each bar represents the mean of 4 biological replicates and error bars indicate the standard error. Different letters indicate significant differences between development stages and locations (p ≤ 0.05). Absence of letters indicates no significant differences between development stages or locations.

## Slide 4
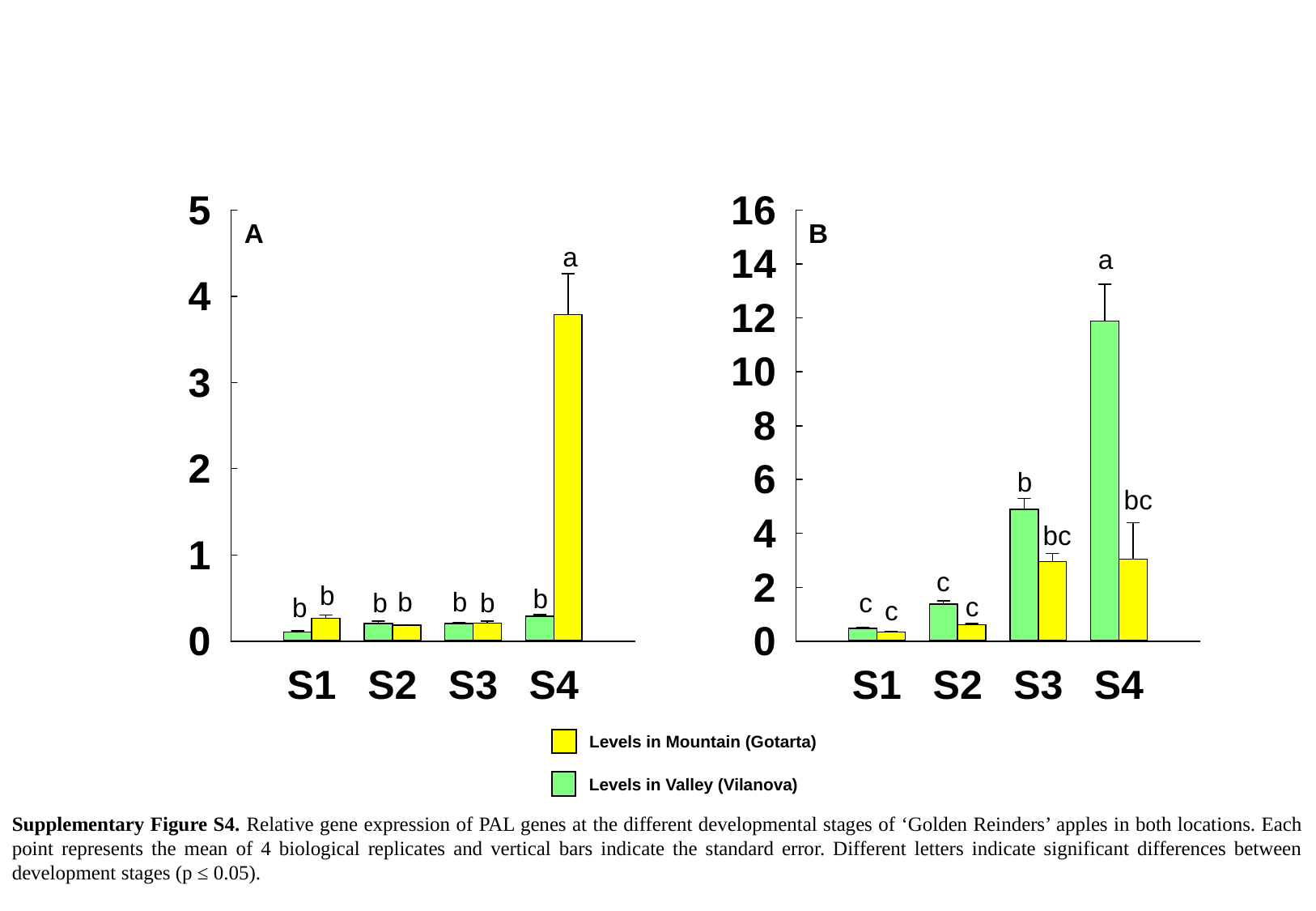

A
B
Levels in Mountain (Gotarta)
Levels in Valley (Vilanova)
Supplementary Figure S4. Relative gene expression of PAL genes at the different developmental stages of ‘Golden Reinders’ apples in both locations. Each point represents the mean of 4 biological replicates and vertical bars indicate the standard error. Different letters indicate significant differences between development stages (p ≤ 0.05).

## Slide 5
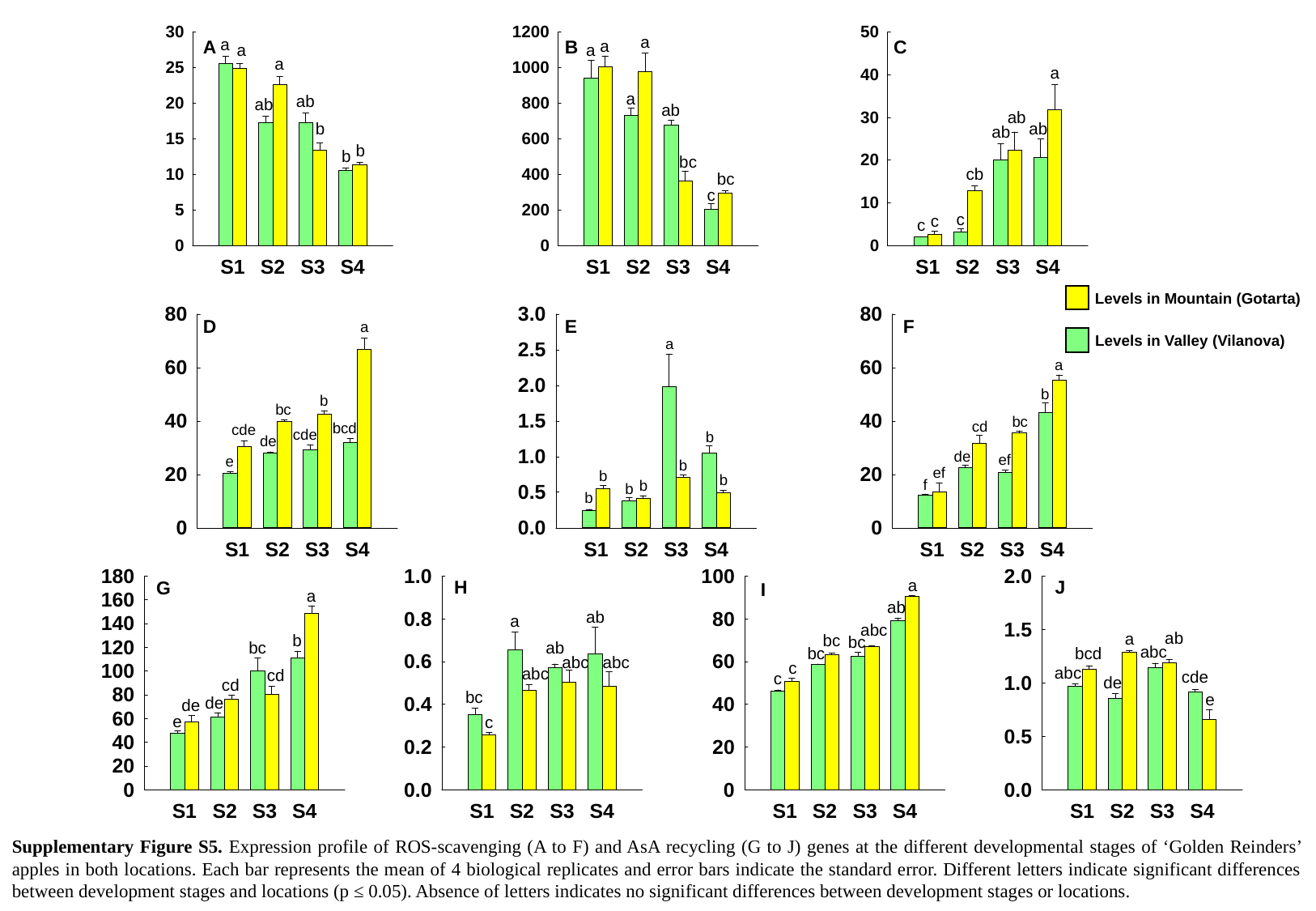

A
B
C
Levels in Mountain (Gotarta)
Levels in Valley (Vilanova)
D
E
F
H
J
G
I
Supplementary Figure S5. Expression profile of ROS-scavenging (A to F) and AsA recycling (G to J) genes at the different developmental stages of ‘Golden Reinders’ apples in both locations. Each bar represents the mean of 4 biological replicates and error bars indicate the standard error. Different letters indicate significant differences between development stages and locations (p ≤ 0.05). Absence of letters indicates no significant differences between development stages or locations.

## Slide 6
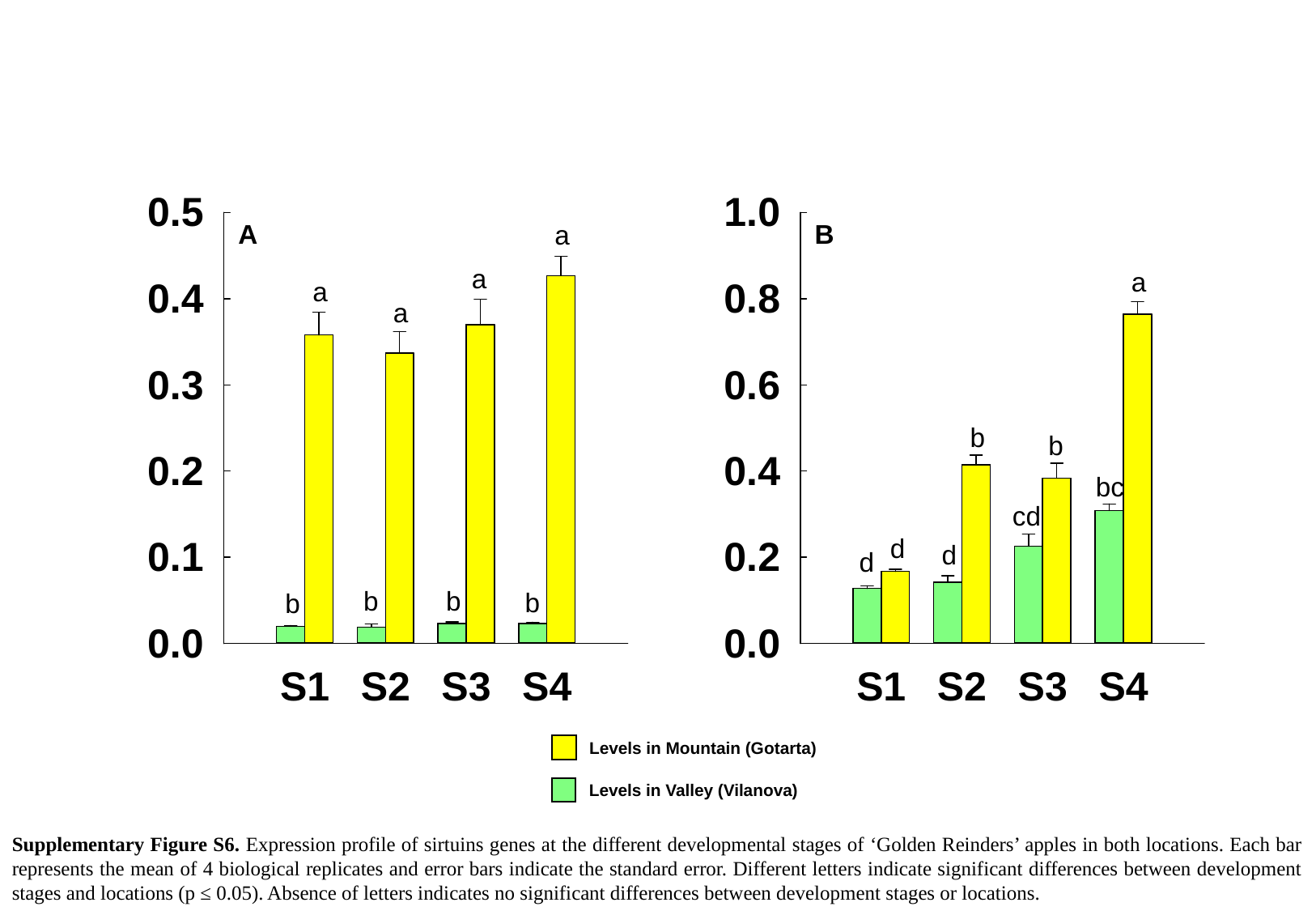

A
B
Levels in Mountain (Gotarta)
Levels in Valley (Vilanova)
Supplementary Figure S6. Expression profile of sirtuins genes at the different developmental stages of ‘Golden Reinders’ apples in both locations. Each bar represents the mean of 4 biological replicates and error bars indicate the standard error. Different letters indicate significant differences between development stages and locations (p ≤ 0.05). Absence of letters indicates no significant differences between development stages or locations.

## Slide 7
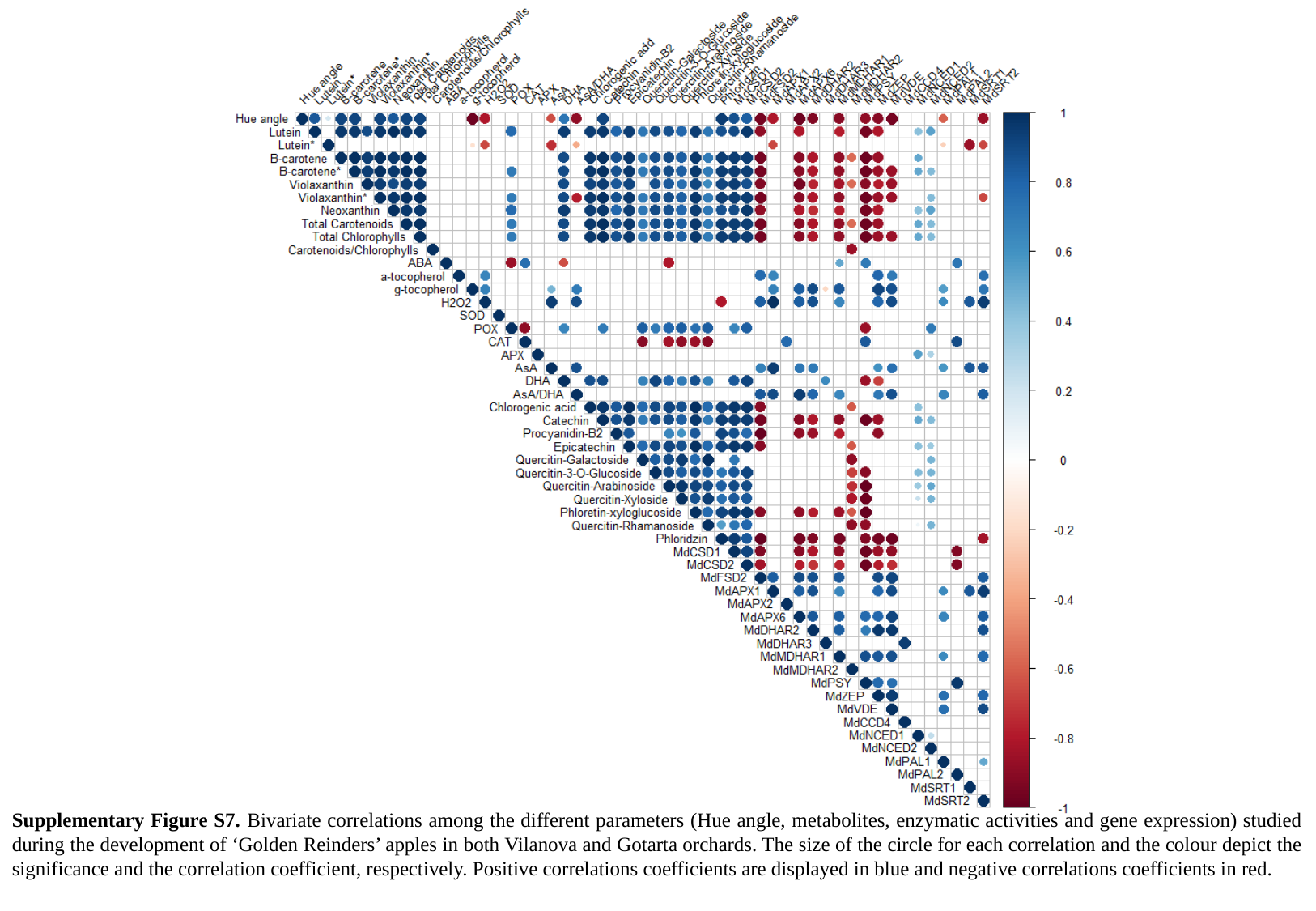

Supplementary Figure S7. Bivariate correlations among the different parameters (Hue angle, metabolites, enzymatic activities and gene expression) studied during the development of ‘Golden Reinders’ apples in both Vilanova and Gotarta orchards. The size of the circle for each correlation and the colour depict the significance and the correlation coefficient, respectively. Positive correlations coefficients are displayed in blue and negative correlations coefficients in red.

## Slide 8
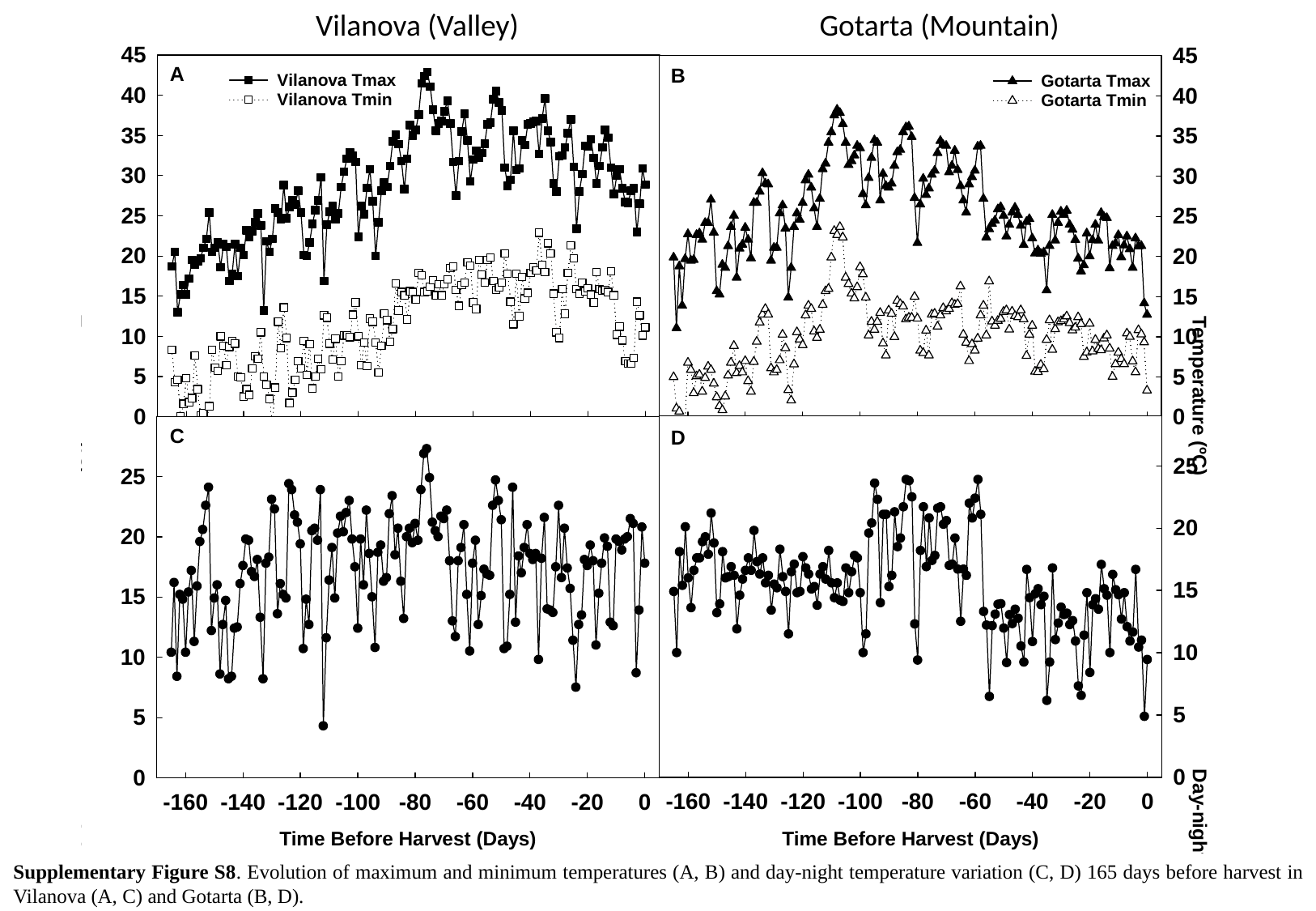

Vilanova (Valley)
Gotarta (Mountain)
A
B
C
D
Supplementary Figure S8. Evolution of maximum and minimum temperatures (A, B) and day-night temperature variation (C, D) 165 days before harvest in Vilanova (A, C) and Gotarta (B, D).

## Slide 9
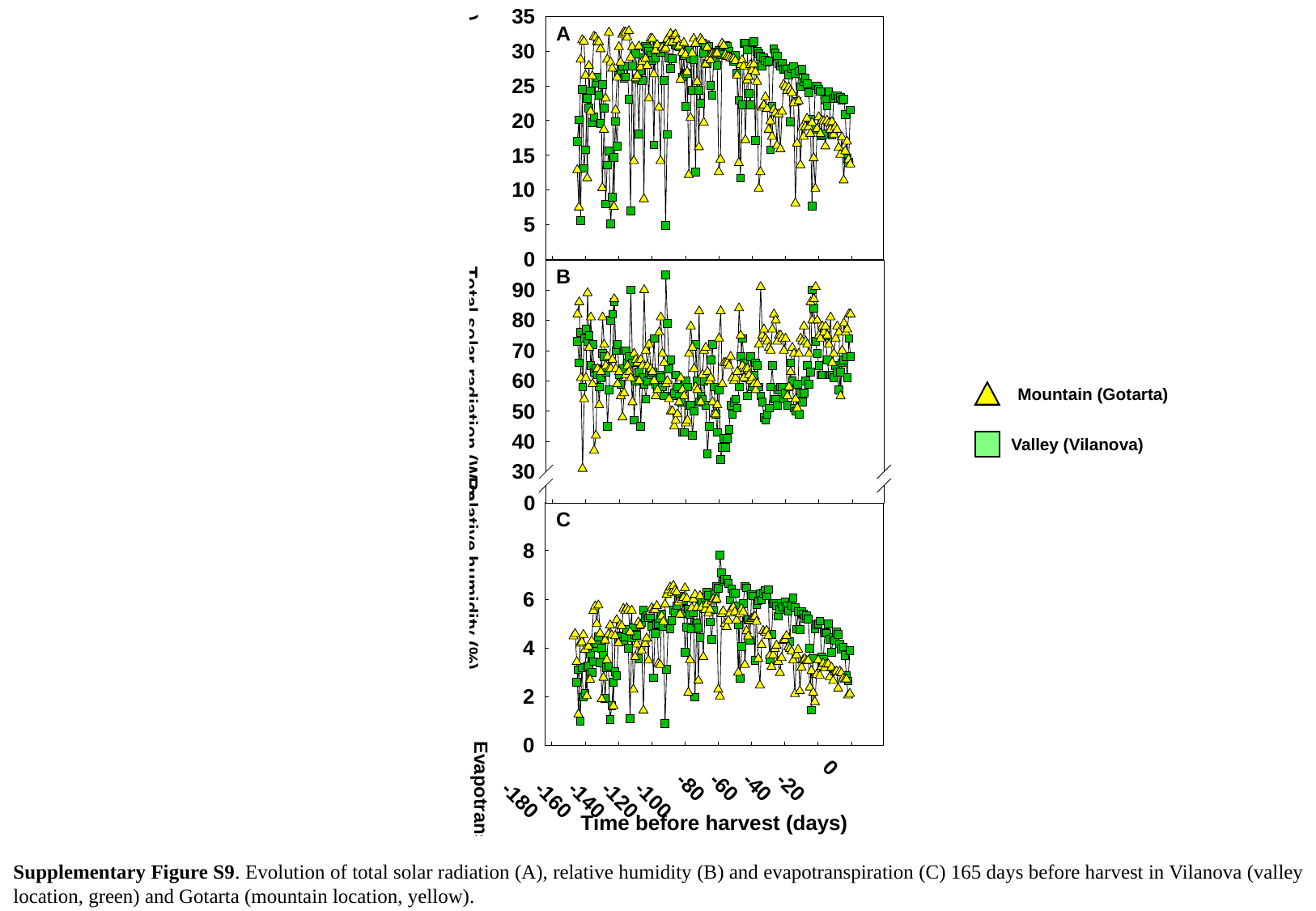

A
B
C
Mountain (Gotarta)
Valley (Vilanova)
Supplementary Figure S9. Evolution of total solar radiation (A), relative humidity (B) and evapotranspiration (C) 165 days before harvest in Vilanova (valley location, green) and Gotarta (mountain location, yellow).

## Slide 10
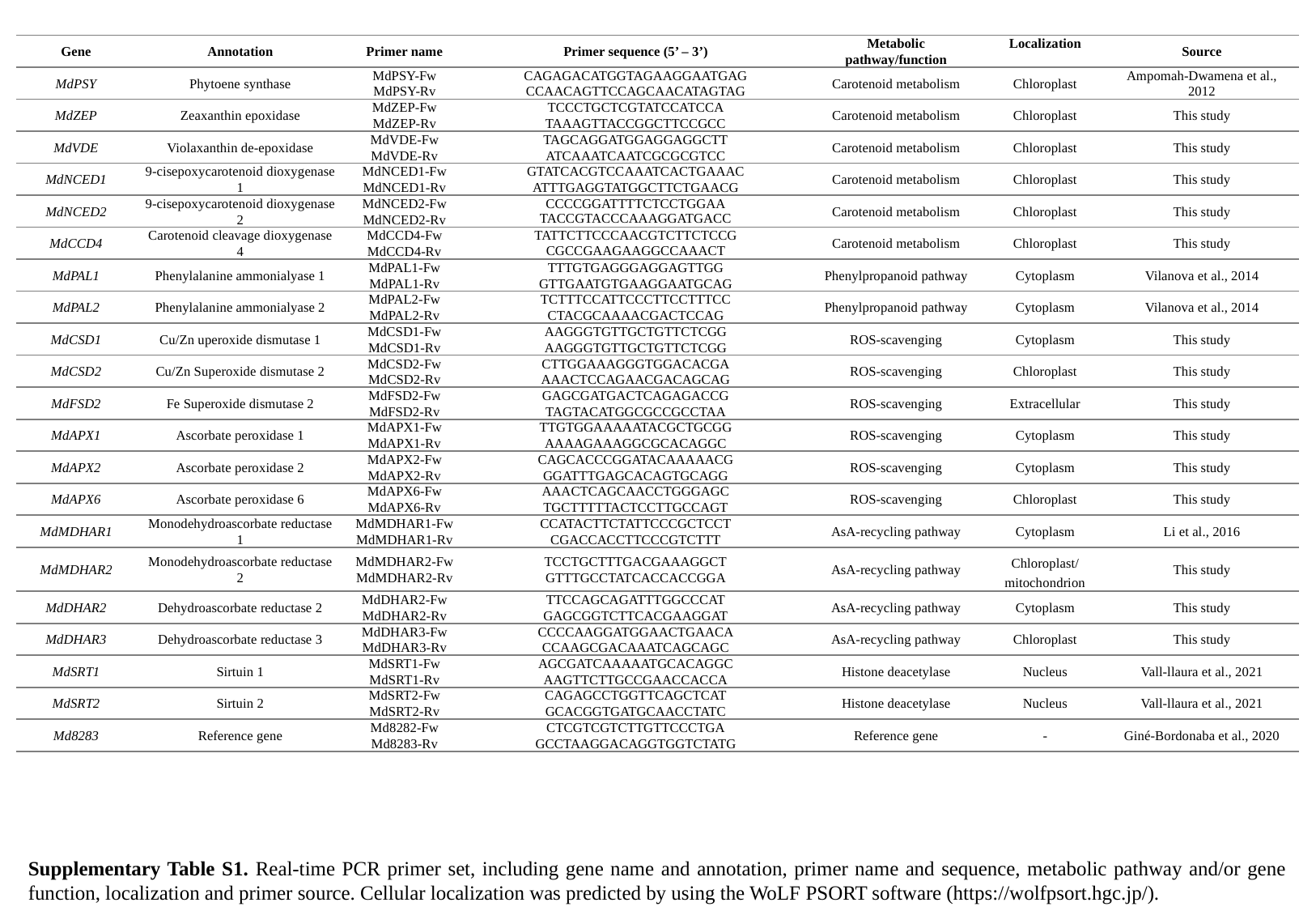

| Gene | Annotation | Primer name | Primer sequence (5’ – 3’) | Metabolic pathway/function | Localization | Source |
| --- | --- | --- | --- | --- | --- | --- |
| MdPSY | Phytoene synthase | MdPSY-Fw MdPSY-Rv | CAGAGACATGGTAGAAGGAATGAG CCAACAGTTCCAGCAACATAGTAG | Carotenoid metabolism | Chloroplast | Ampomah-Dwamena et al., 2012 |
| MdZEP | Zeaxanthin epoxidase | MdZEP-Fw MdZEP-Rv | TCCCTGCTCGTATCCATCCA TAAAGTTACCGGCTTCCGCC | Carotenoid metabolism | Chloroplast | This study |
| MdVDE | Violaxanthin de-epoxidase | MdVDE-Fw MdVDE-Rv | TAGCAGGATGGAGGAGGCTT ATCAAATCAATCGCGCGTCC | Carotenoid metabolism | Chloroplast | This study |
| MdNCED1 | 9-cisepoxycarotenoid dioxygenase 1 | MdNCED1-Fw MdNCED1-Rv | GTATCACGTCCAAATCACTGAAAC ATTTGAGGTATGGCTTCTGAACG | Carotenoid metabolism | Chloroplast | This study |
| MdNCED2 | 9-cisepoxycarotenoid dioxygenase 2 | MdNCED2-Fw MdNCED2-Rv | CCCCGGATTTTCTCCTGGAA TACCGTACCCAAAGGATGACC | Carotenoid metabolism | Chloroplast | This study |
| MdCCD4 | Carotenoid cleavage dioxygenase 4 | MdCCD4-Fw MdCCD4-Rv | TATTCTTCCCAACGTCTTCTCCG CGCCGAAGAAGGCCAAACT | Carotenoid metabolism | Chloroplast | This study |
| MdPAL1 | Phenylalanine ammonialyase 1 | MdPAL1-Fw MdPAL1-Rv | TTTGTGAGGGAGGAGTTGG GTTGAATGTGAAGGAATGCAG | Phenylpropanoid pathway | Cytoplasm | Vilanova et al., 2014 |
| MdPAL2 | Phenylalanine ammonialyase 2 | MdPAL2-Fw MdPAL2-Rv | TCTTTCCATTCCCTTCCTTTCC CTACGCAAAACGACTCCAG | Phenylpropanoid pathway | Cytoplasm | Vilanova et al., 2014 |
| MdCSD1 | Cu/Zn uperoxide dismutase 1 | MdCSD1-Fw MdCSD1-Rv | AAGGGTGTTGCTGTTCTCGG AAGGGTGTTGCTGTTCTCGG | ROS-scavenging | Cytoplasm | This study |
| MdCSD2 | Cu/Zn Superoxide dismutase 2 | MdCSD2-Fw MdCSD2-Rv | CTTGGAAAGGGTGGACACGA AAACTCCAGAACGACAGCAG | ROS-scavenging | Chloroplast | This study |
| MdFSD2 | Fe Superoxide dismutase 2 | MdFSD2-Fw MdFSD2-Rv | GAGCGATGACTCAGAGACCG TAGTACATGGCGCCGCCTAA | ROS-scavenging | Extracellular | This study |
| MdAPX1 | Ascorbate peroxidase 1 | MdAPX1-Fw MdAPX1-Rv | TTGTGGAAAAATACGCTGCGG AAAAGAAAGGCGCACAGGC | ROS-scavenging | Cytoplasm | This study |
| MdAPX2 | Ascorbate peroxidase 2 | MdAPX2-Fw MdAPX2-Rv | CAGCACCCGGATACAAAAACG GGATTTGAGCACAGTGCAGG | ROS-scavenging | Cytoplasm | This study |
| MdAPX6 | Ascorbate peroxidase 6 | MdAPX6-Fw MdAPX6-Rv | AAACTCAGCAACCTGGGAGC TGCTTTTTACTCCTTGCCAGT | ROS-scavenging | Chloroplast | This study |
| MdMDHAR1 | Monodehydroascorbate reductase 1 | MdMDHAR1-Fw MdMDHAR1-Rv | CCATACTTCTATTCCCGCTCCT CGACCACCTTCCCGTCTTT | AsA-recycling pathway | Cytoplasm | Li et al., 2016 |
| MdMDHAR2 | Monodehydroascorbate reductase 2 | MdMDHAR2-Fw MdMDHAR2-Rv | TCCTGCTTTGACGAAAGGCT GTTTGCCTATCACCACCGGA | AsA-recycling pathway | Chloroplast/ mitochondrion | This study |
| MdDHAR2 | Dehydroascorbate reductase 2 | MdDHAR2-Fw MdDHAR2-Rv | TTCCAGCAGATTTGGCCCAT GAGCGGTCTTCACGAAGGAT | AsA-recycling pathway | Cytoplasm | This study |
| MdDHAR3 | Dehydroascorbate reductase 3 | MdDHAR3-Fw MdDHAR3-Rv | CCCCAAGGATGGAACTGAACA CCAAGCGACAAATCAGCAGC | AsA-recycling pathway | Chloroplast | This study |
| MdSRT1 | Sirtuin 1 | MdSRT1-Fw MdSRT1-Rv | AGCGATCAAAAATGCACAGGC AAGTTCTTGCCGAACCACCA | Histone deacetylase | Nucleus | Vall-llaura et al., 2021 |
| MdSRT2 | Sirtuin 2 | MdSRT2-Fw MdSRT2-Rv | CAGAGCCTGGTTCAGCTCAT GCACGGTGATGCAACCTATC | Histone deacetylase | Nucleus | Vall-llaura et al., 2021 |
| Md8283 | Reference gene | Md8282-Fw Md8283-Rv | CTCGTCGTCTTGTTCCCTGA GCCTAAGGACAGGTGGTCTATG | Reference gene | - | Giné-Bordonaba et al., 2020 |
Supplementary Table S1. Real-time PCR primer set, including gene name and annotation, primer name and sequence, metabolic pathway and/or gene function, localization and primer source. Cellular localization was predicted by using the WoLF PSORT software (https://wolfpsort.hgc.jp/).
